# Modified Carbon Nanotubes Favor Fibroblast Growth by Tuning the Cell Membrane Potential

**DOI:** 10.1021/acsami.3c14527

**Published:** 2024-01-11

**Authors:** Giulia Suarato, Samuel Pressi, Enzo Menna, Massimo Ruben, Enrica Maria Petrini, Andrea Barberis, Dalila Miele, Giuseppina Sandri, Marco Salerno, Andrea Schirato, Alessandro Alabastri, Athanassia Athanassiou, Remo Proietti Zaccaria, Evie L. Papadopoulou

**Affiliations:** † Istituto Italiano di Tecnologia, via Morego 30, 16163 Genova, Italy; ‡ Department of Chemical Sciences, University of Padua, via Marzolo 1, 35131 Padova, Italy; § Interdepartmental Centre Giorgio Levi Cases for Energy Economics and Technology, University of Padua, via Marzolo 9, 35131 Padova, Italy; ∥ Department of Drug Sciences, University of Pavia, via Taramelli 12, 27100 Pavia, Italy; ⊥ Dipartimento di Fisica, Politecnico di Milano, Pizza Leonardo da Vinci 32, Milan 20133, Italy; # Department of Electrical and Computer Engineering, Rice University, 6100 Main Street, Houston, Texas 77005, United States

**Keywords:** electrospun fibers, polarization fields, membrane potential, PLA fibers, carbon nanotube functionalization, fibroblast electrophysiology, nonexcitable cells, PLA-carbon nanotube composites

## Abstract

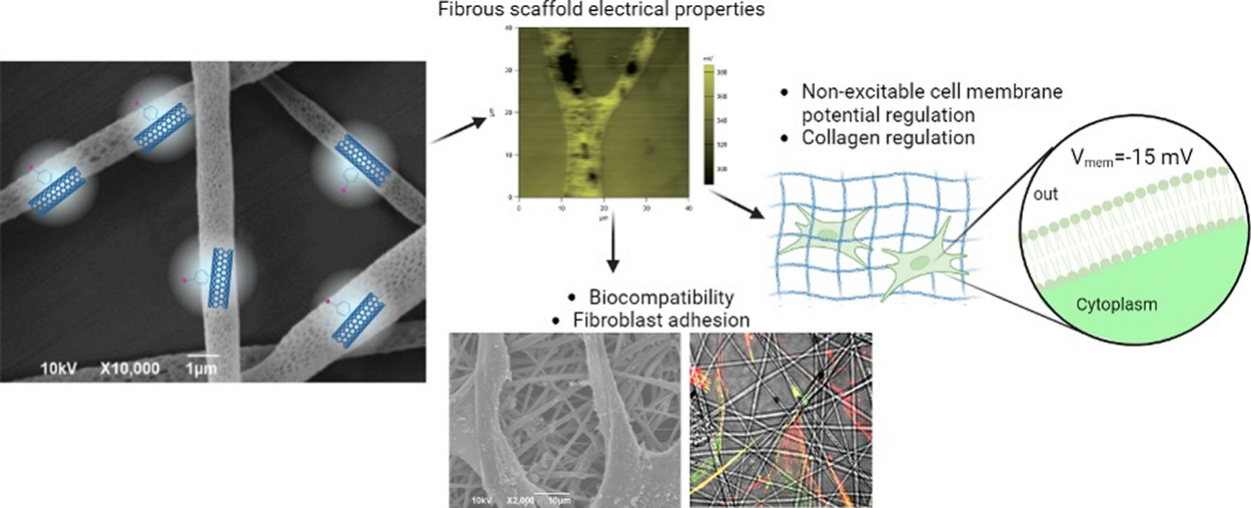

As is known, carbon nanotubes favor cell growth in vitro, although the underlying mechanisms are not yet fully elucidated. In this study, we explore the hypothesis that electrostatic fields generated at the interface between nonexcitable cells and appropriate scaffold might favor cell growth by tuning their membrane potential. We focused on primary human fibroblasts grown on electrospun polymer fibers (poly(lactic acid)—PLA) with embedded multiwall carbon nanotubes (MWCNTs). The MWCNTs were functionalized with either the *p*-methoxyphenyl (PhOME) or the *p*-acetylphenyl (PhCOMe) moiety, both of which allowed uniform dispersion in a solvent, good mixing with PLA and the consequent smooth and homogeneous electrospinning process. The inclusion of the electrically conductive MWCNTs in the insulating PLA matrix resulted in differences in the surface potential of the fibers. Both PLA and PLA/MWCNT fiber samples were found to be biocompatible. The main features of fibroblasts cultured on different substrates were characterized by scanning electron microscopy, immunocytochemistry, Rt-qPCR, and electrophysiology revealing that fibroblasts grown on PLA/MWCNT reached a healthier state as compared to pure PLA. In particular, we observed physiological spreading, attachment, and *V*_mem_ of fibroblasts on PLA/MWCNT. Interestingly, the electrical functionalization of the scaffold resulted in a more suitable extracellular environment for the correct biofunctionality of these nonexcitable cells. Finally, numerical simulations were also performed in order to understand the mechanism behind the different cell behavior when grown either on PLA or PLA/MWCNT samples. The results show a clear effect on the cell membrane potential, depending on the underlying substrate.

## Introduction

Living cell functions are highly dependent on the extracellular environment. The biophysical relationship of a cell with its extracellular environment is a dynamic one, as several extracellular stimuli can affect or even define the cell fate.^1^ In recent years, accumulating evidence has demonstrated that extracellular stimuli conveyed to the cells via the cell/material interface can shape cellular responses and, ultimately, influence fundamental functions, such as adhesion, growth, proliferation, and differentiation. In this framework, cutting-edge manufacturing techniques that enable the design, fabrication, and functionalization of materials at the nanoscale have been instrumental, since materials engineering allows the fabrication of scaffolds with properties that act as extracellular stimuli (i.e., surface topography, spatially defined biochemical cues, stiffness gradient, etc.).^1−3^

The first evidence that electrical signals can significantly modify biological systems dates back to 1947 when Marsh and Beams showed that the application of an external electric field could change the polarity of regenerating fragments in *Dugesia tigrina*.^4,5^ Electrical stimulation in cell cultures^6−8^ has been applied mainly to excitable cells, i.e., neurons or cardiomyocytes. In those conditions, technological advancements have produced electroactive scaffolds that will serve either as electrodes for recordings or to deliver electrical signals for advanced tissue engineering.^9−17^ Furthermore, in the last couple of decades, it has been established that the application of external electric fields plays a critical role also in wound healing, even though this physiological tissue restoration process mainly involves nonexcitable cells (e.g., fibroblasts, keratinocytes, macrophages, endothelial cells, and blood cells).^18−21^ To this end, fibroblasts grown on electrically conductive substrates have shown enhanced adhesion and metabolic activity upon electrical stimulation, leading to increased proliferation.^22−24^

The cell membrane wraps cells with a double layer of lipid molecules, in which carbohydrates and proteins are embedded. Among them, ionic pumps and channels generate a constant flow of different ions (bioelectricity), giving rise to an electrical charge imbalance between the interior and the exterior sides of the cell membrane and a consequent potential difference called transmembrane potential, *V*_mem_.^25^*V*_mem_ was traditionally correlated with cell excitability; however, in the last years, its central importance for vital cell functions has been recognized as *V*_mem_ is associated with cell cycle, cell volume, proliferation, differentiation,^25−27^ or as an instructing patterning cue for large-scale anatomy,^28^ also for nonexcitable cells. Specifically for fibroblasts, pharmacological depolarization of *V*_mem_ leads to proliferation.^29^ Interfacing neuronal^30^ and bacterial cells^31^ with charged nanomaterials results in changes in their surface charge distribution and in their electrical properties. Recently, we have demonstrated that interfacial electrostatic fields affect the *V*_mem_ of glioblastoma stem-like cells, triggering their differentiation to the glial phenotype.^32^ In this frame, the modulation of *V*_mem_ represents a fundamental control mechanism of various biological functions, rendering it a powerful tool for the design of new drugs and strategies for regenerative tissue engineering and stem cell therapies.

PLA is a biocompatible insulating material that has been widely used to fabricate scaffolds for a plethora of cell cultures. It can be easily filled with multiwall carbon nanotubes (MWCNTs) in order to obtain electroactive materials.^33^ MWCNTs covalently functionalized with *p*-methoxyphenyl moieties (MWCNT-PhOMe) can be homogeneously dispersed in a PLA matrix to obtain composite films^34^ or electrospun fibers,^35^ which promote neuronal growth and differentiation of human neuroblastoma cells (SH-SY5Y)^34,35^ and human circulating multipotent stem cells from peripheral blood.^36,37^ However, the hypothesis that surface polarization might favor the growth of nonexcitable cells by tuning the cell membrane potential is a new concept that has not been deeply investigated.

Herein, we have fabricated PLA electrospun fibers with surface polarization fields stemming from embedded derivatives of MWCNTs and we have studied their effect on the membrane potential and the growth of primary human fibroblasts. In particular, we fabricated PLA composite electrospun fibers with MWCNT, bearing either *p*-methoxyphenyl (PhOME) or *p*-acetylphenyl (PhCOMe) functional groups, which provide solubility and allow homogeneous dispersion in polymer matrices. The variation of the electrical charge distribution between the insulating PLA and the electrically conductive MWCNTs on the fiber surface potential was measured by the Kelvin probe mode of an atomic force microscope (AFM). We also demonstrated that the presence of electrically conductive MWCNTs determines morphological and functional changes in primary human dermal fibroblasts, as observed in confocal imaging and electrophysiology experiments, after 3 days in vitro (DIV3). Finally, the electrostatic response of the system comprising the fibroblasts grown either on PLA or on the composite scaffolds was numerically simulated, revealing the direct correlation between charged scaffolds and resulted electrostatic fields at the cell/material interface and the cell membrane potential, a parameter that is known to strongly influence cell growth. Our findings highlight how, besides the biocompatibility of an inert polymer matrix (used pristine for decades in the biomedical fields), the tuning of its electrical features by means of advanced, conductive nanofillers is central in shaping a more appropriate interface for cell growth and, ultimately, tissue development.

## Experimental Methods

### Materials

Purified MWCNTs (OD < 8 nm, ID 2–5 nm, length 9.5–2 μm) were purchased from ACS Materials. PTFE membranes (0.2 μm, 47 mm, FluoroporeTM) were purchased from Merck Millipore. *N*-Cyclohexyl-2-pyrrolidone (CHP, 99% purity), isopentyl nitrite (96–98% purity), 4′-aminoacetophenone (99% purity), and 4-methoxyaniline (99% purity) were purchased from Merck. All reagents and solvents were used as received.

Poly(lactic acid) (PLA) was purchased from Nature Works (6060D). Chloroform (CHCl_3_) and dimethylformamide (DMF) were purchased from Sigma-Aldrich and used as received.

### Synthesis of MWCNT-PhOMe

We followed the procedure from our previous publications.^31,37^ In short, MWCNTs (10 mg, 0.83 mmol of C) were dispersed into 7 mL of CHP by pulsed microtip sonication for 10 min (Titanium tip Misonix 3000 sonicator, intensity level 2.0:4–6 W, interval 3 s on and 3 s off) and transferred into a 250 mL two-neck round-bottom flask. 4-Methoxyaniline (51.3 mg, 0.415 mmol, 0.5 equiv) was dissolved in 3 mL of CHP and added to the dispersion. The reaction mixture was heated to 80 °C fluxing N_2_ under magnetic stirring. Then, isopentyl nitrite (56 μL, 0.415 mmol, 0.5 equiv) was added. After 15 min, the reaction mixture was cooled down, and 100 mL of cold methanol was added. The obtained dispersion was filtered on a PTFE membrane (0.2 μm), and the solid product was washed with 2 × 100 mL of methanol. The functionalized MWCNTs were removed from the filter through sonication in methanol. The dispersion was centrifugated (4000 rpm for 10 min) and the solid was recovered and dried under an IR lamp for 1 h.

### Synthesis of MWCNT-PhCOMe

MWCNTs (10 mg, 0.83 mmol of C) were dispersed into 7 mL of CHP by pulsed microtip sonication for 10 min (Titanium tip Misonix 3000 sonicator, intensity level 2.0:4–6 W, interval 3 s on and 3 s off) and transferred into a 250 mL two-neck round-bottom flask. 4′-Aminoacetophenone (56.1 mg, 0.415 mmol, 0.5 equiv) was dissolved in 3 mL of CHP and added to the dispersion. The reaction mixture was heated to 80 °C fluxing N_2_ under magnetic stirring. Then, isopentyl nitrite (56 μL, 0.415 mmol, 0.5 equiv) was added. After 15 min, the reaction mixture was cooled down and 100 mL of cold methanol was added. The obtained dispersion was filtered on a PTFE membrane (0.2 μm), and the solid product was washed with 2 × 100 mL of methanol. The functionalized MWCNTs were removed from the filter through sonication in methanol. The dispersion was centrifugated (4000 rpm for 10 min) and the solid was recovered and dried under an IR lamp for 1 h.

### Electrospinning

PLA was dissolved in a CHCl_3_:DMF solution at a volume ratio of 8:2 and a concentration of 10% polymer in the solution. In the case of composite fibers, 1 wt % of MWCNT was added to the solution and sonicated overnight to achieve homogeneity. Electrospinning was carried out using a vertical setup. The solutions were inserted in a syringe and connected to a syringe pump (NE-1000 New Era Pump Systems, Inc.). An 18-gauge, stainless-steel needle was used as a spinneret, clumped to the positive electrode of a high-voltage power supply (EH40R2.5, Glassman High Voltage, Inc.), while the aluminum collector (64 cm^2^), placed at 23 cm from the spinneret tip, was grounded. The solutions could be electrospun when a voltage of 18–20 kV was applied at a flow rate of 200–600 μL/h.

### Scanning Electron Microscopy

The morphology of the electrospun fibers was analyzed by scanning electron microscopy (SEM; JSM-6490L; Jeol, Tokyo, Japan). The samples were coated with 10–15 nm of gold before imaging, using a sputter coater.

### Raman Spectroscopy

Micro-Raman spectra were obtained in ambient conditions using a Horiba Jobin-Yvon LabRAM HR800 μRaman spectrometer equipped with a microscope. In order to excite the samples, a 632.8 nm excitation line was used in backscattering geometry through a 50× objective lens. The power used was 0.25 mW. The grating was of 600 lines per mm with a spectral resolution of ∼1 cm^–1^.

### Wetting Properties

The static contact angle was measured by an automated tension meter (OCAH-200 DataPhysics Germany) using the sessile drop method. A syringe placed 5 μL on the sample, and the WCA was measured after 10 s.

### Atomic Force Microscopy and Kelvin Probe

Scanning Kelvin probe microscopy is a variant of atomic force microscopy (AFM). During the measurements, first, the AFM was operating in tapping mode to track the topography. After each line, the tip was moving up reaching a distance Δ*H* over the previously mapped surface (lift or nap pass) and was scanned at this height following the previously acquired surface profile in order to maintain the distance constant. During this second scan of the same line, the surface potential was detected according to the Kelvin probe principle: tip and sample surface are the plates of a capacitor, and the force due to the electric field between the plates is canceled out by a feedback loop, which adds to the tip a DC voltage that is the same as the surface potential (while the highly doped silicon sample substrate is set to ground). An MFP 3D (Asylum Research, CA, USA) AFM was used for these measurements, with a MESP probe (Bruker, MA, USA), having nominal resonance frequency and tip apex diameter of 75 kHz and 70 nm, respectively, the latter being due to a ∼25 nm thick metal (CoCr) coating, necessary for electrical polarization of the tip “plate”. The maps were acquired with a 40 × 40 μm^2^ scan area (corresponding to 256 × 256 pixels) with 0.5 Hz line frequency; the elevation height for the nap pass was Δ*H* = 100 nm.

### Cell Plating

Adult primary human dermal fibroblasts (HDFa, Thermo Fisher Scientific) were used as our *in vitro* model. Cells were cultured in T75 culture flasks in the presence of Fibroblast Growth Medium 2 (Sigma-Aldrich) supplemented with a supplement pack containing fetal calf serum (0.02 mL/mL), basic fibroblast growth factor (recombinant human, 1 ng/mL), and insulin (5 μg/mL) in an incubator at 37 °C and with 5% CO_2_, until reaching confluency. Cells were cultured on PLA and PLA/MWCNTs electrospun fibers. Bare glass coverslips were used as the control substrates. In addition, with the dual aim of reducing the hydrophobicity of the fibrous matrices and assessing the effect of an extracellular matrix coating on the overall cell adhesion, a set of samples were coated with FN (Fibronectin Human Protein, Native, Thermo Fisher Scientific). The samples were incubated with 400 μL of FN at a concentration of 20 μg/mL for 1.5 h in a humidified incubator at 37 °C and with 5% CO_2_. Primary human adult dermal fibroblasts were used at early passages (P2–P6), and seeded at different densities, depending on the type of experiment, as described later. Cells were allowed to attach and grow onto the fibrous network or onto the glass coverslips in the presence of Fibroblast Growth Medium 2 (Sigma-Aldrich) supplemented with a supplement pack containing fetal calf serum (0.02 mL/mL), basic fibroblast growth factor (recombinant human, 1 ng/mL), and insulin (5 μg/mL) for 72 h in a humidified incubator at 37 °C and with 5% CO_2_.

### SEM of Cell Cultures

Fibrous mats were cut in circular shapes in order to fit the wells of a 24-well plate and sterilized under ultraviolet light for 30 min (15 min per side). A PDMS ring was inserted in each well to prevent the fibrous matrix from floating during the experiment. Cell plating took place as described above, with a cell density of 5000 cells/cm^2^. The samples were subsequently prepared as in Suarato et al.^38^ Briefly, the fibroblast-plated fibrous scaffolds were fixed in a solution of 2% glutaraldehyde in 0.1 M cacodylate buffer for 2 h at room temperature (RT). After several washes in the same buffer, the samples were postfixed in osmium tetroxide (1% in Milli-Q water) for 2 h and washed with Milli-Q water. Afterwards, the samples were dehydrated with a series of incubations in ethanol/water solutions of increasing concentrations (30–100%, 10 min each), followed by incubation in 1:1 ethanol:hexamethyldisilazane (HMDS, Sigma-Aldrich) and 100% HMDS. The dehydrated samples were air-dried overnight. The samples were coated with 10–15 nm of gold before SEM imaging, using a sputter coater.

### FAK Staining

The focal adhesion kinase (FAK) staining was performed by immunolabeling vinculin in cultured fibroblasts. Briefly, PLA and PLA/MWCNTs electrospun fibers were deposited onto squared glass coverslips (22 × 22 mm^2^) in order to fit the well of a 6-well plate and sterilized under ultraviolet light for 20 min. Cell plating took place as described above, with a cell density of 5000 cells/cm^2^. To allow the cytoskeleton actin staining, samples underwent permeabilization with 0.1% Triton X-100 for 8 min and then washed twice with prewarmed PBS. To limit unspecific binding of the primary antibody, samples were immersed in a blocking solution (10% normal goad serum, Abcam, in PBS) for 1 h at RT and then washed twice with prewarmed PBS. The primary antibody (vinculin monoclonal antibody, Sigma-Aldrich) was prepared in the blocking solution at 1:400 dilutions and incubated for 1 h at RT. Subsequently, samples were washed three times with prewarmed PBS (5 min each time) and incubated in the staining solution containing the secondary antibody (AlexaFluor488 Goat Anti-Mouse (IgG), Abcam, 1:1000 dilutions in 1% bovine serum albumin), and the actin fibrils marker (AlexaFluor546 Phalloidin, Thermo Fisher Scientific, 1:100 dilutions in 1% bovine serum albumin). The incubation was carried out for 1 h at RT, in the dark. Subsequently, samples were washed three times with prewarmed PBS (5 min each time) and incubated in a DAPI solution for nuclei staining (Thermo Fisher Scientific, 2.5 μg/mL in 1% bovine serum albumin) for 15 min, in the dark. The stained samples were mounted with Fluoromont-G onto a glass slide and imaged with a Nikon A1 confocal microscope equipped with 405, 488, and 560 nm lasers.

### Cell Morphology Analysis

The morphology of fibroblasts plated onto the different substrates involved was quantified as the “cell area” and “circularity index” in confocal images analyzed using the ImageJ software (https://imagej.nih.gov). Briefly, after a first color balance adjustment, single, isolated cells were cropped via the “freehand” selection button; their threshold was adjusted to identify the complete cell contours, and the cell area was obtained via the Analyze Particle plugin. The same analysis parameters were applied to all images. Moreover, lines were drawn along the major and minor axes of the cells and measured; the circularity index was calculated as the ratio between the minor axis and the major one. A statistical analysis of the data was performed in GraphPad via one-way ANOVA followed by Bonferroni’s posthoc test [considering significant *p* values <0.05 (*), 0.01 (**), and 0.001 (***)].

### FAK Quantitative Analysis

FAK quantitative analysis was performed on confocal images of fibroblasts plated onto the different substrates to quantify vinculin trait number and length. By using the ImageJ software (https://imagej.nih.gov) the cell area was initially derived as described above. Then, with the “point tool” and the “straight line tool” the vinculin traits were counted and measured and a series of parameters were considered: average number of traits per cell, average length of traits per cell, number of traits/cell area (also named “normalized number of traits”), and total length of traits/cell area (also named “normalized length of traits”). A statistical analysis of the data was performed in GraphPad via one-way ANOVA followed by a Bonferroni’s posthoc test (parametric comparison) or a Kruskal–Wallis posthoc test (nonparametric comparison), considering significant *p* values <0.05 (*), 0.01 (**), and 0.001 (***).

### Fiber Extract Cytocompatibility

Fibroblasts were seeded onto 24-well plates at a density of 7000 cells/cm^2^ and allowed to attach overnight. The extraction media from the fibrous matrices were prepared following the ISO10993-12_2009 standard test specifications. Briefly, the samples were cut into 6 cm^2^ pieces, placed into 35 mm Petri dishes, and sterilized under UV light for 20 min (10 min per side). One milliliter of culture medium was added to each dish to wet the fibers and incubation at 37 °C was carried out for 24 h. The following morning attached HDFa cells were treated with 0.5 mL of the extraction media and incubated for an additional 24 or 48 h, while cells incubated in a normal growth medium were considered as controls. To determine fibroblast viability, an MTS assay (tetrazolium salt, CellTiter 96AQ_ueous_ One Solution Cell Proliferation Assay, Promega) was conducted following the protocol previously established in our group.^38^ Results are reported as the mean ± standard error. A Student’s *t*-test, assuming unequal variances, was carried out, considering a value *p* < 0.01 as significant. With the aim of visualizing the morphology of the cells exposed to the PLA and PLA/MWCNT fibers extracts, fibroblasts were plated onto glass coverslips at a density of 5000 cells/cm^2^ and treated as reported above. After 24 h of treatment, cells were washed with prewarmed PBS (pH 7.4) and fixed with 3.7% paraformaldehyde (PFA) for 20 min. Nuclei staining was obtained via DAPI incubation (2.5 μg/mL in PBS) for 15 min in the dark, followed by two washing with PBS. The samples were then permeabilized with 0.3% Triton X-100 for 8 min and washed twice with PBS, prior to incubation in Alexa Fluor 488 Phalloidin (Thermo Fisher Scientific, 1:100 dilutions in PBS) for 20 min in the dark. The coverslips were mounted with Fluoromont-G onto glass slides and imaged under a confocal microscope Nikon A1 (equipped with 405 and 488 lasers).

### Live/Dead Staining

PLA and PLA/MWCNTs electrospun fibers were deposited onto 13 mm round glass coverslips; they were sterilized under UV light for 20 min and placed at the bottom of a 24-well plate. HDFa cells were seeded at a density of 7000 cells/cm^2^ and cultured as described above. At the desired time points, 1.5 μL of calcein-AM (4 mM solution in DMSO, Sigma-Aldrich) and 1 μL of ethidium homodimer (2 mM solution in DMSO, Sigma-Aldrich) were added in each well (containing 500 μL of supplemented media) and incubated for an additional 45 min in a humidified chamber at 37 °C and with 5% CO_2_. Images were taken immediately after the incubation by means of a confocal microscope Nikon A1, equipped with a 20× objective, and with 488 and 401 nm lasers. An average of 30 images per sample were acquired and used for the cell counting, performed on ImageJ (https://imagej.nih.gov) via the *Cell Counter* plugin. Results are reported as mean ± standard error. A Student’s *t*-test, assuming unequal variances, was carried out, considering a value *p* < 0.01 as significant.

### PCR Analysis

RT-qPCR was performed to investigate the *in vitro* differences in expression of extracellular matrix protein (collagen) and proliferation/apoptotic stimulus (Bcl-2 and Bax) of HDFa grown onto PLA and PLA/MWCNTs electrospun fibers, at DIV3. Rt-qPCR was conducted on a total of three replicates per time point. The total RNA was isolated with TriZol agent (Thermo Fisher Scientific, Italy) according to the manufacturer’s instructions and quantified spectrophotometrically at 260 nm by means of a FLUOstar Omega Microplate Reader (FLUOstar Omega—BMG LabTech, D) equipped with a L-Vis microplate. One μg of RNA was used as a template for the synthesis of the cDNA and reverse transcription was carried out using the SimpliAmp Thermal Cycler, following the manufacturer’s instructions of the iScript cDNA Synthesis Kit (BioRad, Milan, Italy). Primers for detecting gene expression of Col1a, Bax, and Bcl-2 were designed by Biorad (Biorad, Milan, Italy). PCR solution (20 μL) was composed of 1 μLcDNA (25 ng), 10 μL of master mix solution of SsoAdvanced Universal SYBR Green Supermix (Biorad, Milan, Italy), and 1 μL of each primer. The ΔΔ*Ct* method was used for data analysis, and the gene expressions were normalized for the housekeeping gene GAPDH. The sequences and the amplicon length of the primers involved in the study are listed in Table S1. The thermal cycling program was performed by means of a StepOnePlus Real-Time PCR System and set as follows: polymerase was activated in 30 s at 95 °C; subsequently, the DNA denaturation was reached in 15 s at 95 °C and the annealing step occurred at 60 °C for 30 s. Denaturation and annealing cycles were repeated 40 times. Finally, melt curves were recorded.

### Electrophysiology

PLA and PLA/MWCNTs electrospun fibers deposited onto 18 mm round glass coverslips were sterilized under the UV light for 20 min and placed at the bottom of a 12-well plate at a density of 1500 cells/cm^2^, where they were allowed to attach and grow on the fibers for 24 and 72 h, as described above. Cells plated onto fibronectin-coated (Fibronectin Human Protein, Native, Thermofisher, 20 μg/mL in PBS for 1.5 h at 37 °C) coverslips were considered as control samples. Resting membrane potentials were recorded in the whole-cell configuration of the patch-clamp technique at RT. The external recording solution contained (in mM) the following: 145 NaCl, 2 KCl, 2 CaCl_2_, 2 MgCl_2_, 10 glucose, and 10 HEPES, pH 7.4. Patch pipettes, pulled from borosilicate glass capillaries (Warner Instruments, LLC, Hamden, USA) had a 4–5 MΩ resistance when filled with intracellular solution. In all experiments, the intracellular solution contained (in mM): 10 KGluconate, 125 KCl, 1 EGTA, 10 HEPES, 5 sucrose, 4 MgATP (300 mOsm and pH 7.2 with KOH). Membrane potentials were recorded using Clampex 10.0 software (Molecular Devices, Sunnyvale, CA). During the cell-attach configuration, a seal resistance of at least 1 GΩ in every cell was reached before the membrane opening. The stability of the patch was checked by monitoring the input resistance during the experiments to exclude cells exhibiting more than 15% changes from the analysis.

### Simulations

To simulate numerically the electrostatic response of the system presented in Figure 6, a finite element method (FEM)-based two-dimensional (2D) model was developed using commercial software (COMSOL Multiphysics 6.0). The simulation includes two segregated calculations, solving for Poisson’s equation in two conditions. In the first step of the computation, serving as a reference, the system consists of a cell on PLA only, and the electrostatic problem is solved by fixing to 0 V (6 meV) the potential at the inner (outer) surface of the cell membrane. No charge is defined in the domain at this stage of the calculation. With the solution of the first step at hand, a second calculation is performed in cascade. This further simulation is formally equivalent to the previous where boundary conditions are appropriately modified to account for the change in the cell substrate (PLA/MWCNTs instead of pure PLA). Specifically, charge densities are defined at (i) the inner and outer surfaces of the membrane and set equal to their corresponding values estimated from the solution of the first step; (ii) the surface of the PLA/MWCNT fiber (refer to the schematic of the considered numerical geometry in Figure 6a) that, instead, modify the electric potential difference along the membrane. No potential is defined in this second step, as its values are the results of the calculations that consider the additional surface charges. Note that fixing the charge densities along the membrane surfaces to the values solved for in the first step ensures that the membrane exhibits the same electrostatic properties as the previous step (that is, without adding a charge on the fiber, the second step provides with the same electric potential results as the first). For both calculations, the cell and its substrate are surrounded by a 30 μm × 30 μm squared domain (the electrolyte), and infinite element (IE) domains are defined at the boundaries of the numerical domain to truncate the calculation and mimic an effective extended simulation domain, and the potential is set to ground (0 V) beyond IEs.

## Results and Discussion

Initially, MWCNTs were functionalized following a modified procedure based on Tour reaction,^39^ starting from aniline derivatives that, in the presence of isopentyl nitrite, give an in situ diazotization reaction on the CNT surface to afford MWCNT-PhOMe and MWCNT-PhCOMe, as depicted in Figure S1a (SI). The electrical properties of MWCNTs can be inhibited by a massive functionalization introducing sp^3^ defects in the sp^2^ carbon lattice and shielding the nanotube surface with an organic insulating layer.^40^ Consequently, reaction conditions were optimized to grant a sufficient functionalization degree (FD), defined as the fraction of carbon atoms of MWCNT that are functionalized, while retaining the native properties of MWCNTs.^41^ Raman spectroscopy (Figure S1b, SI) confirmed that the structural integrity of the MWCNTs was preserved after functionalization. As evaluated by thermogravimetric analysis^41^ (Figure S1c, SI), the FD was 0.42 and 0.67 for MWCNT-PhOMe and MWCNT-PhCOMe, respectively. Such a difference in FD does not affect their solubility in chloroform, whereas nonfunctionalized MWCNT could not form stable solutions. Therefore, the specific derivatives were selected, among several that bear different organic moieties, based on their solubility in chloroform, which is pivotal for a smooth electrospinning process, resulting in regular and homogeneous fibers.

**Figure 1:**
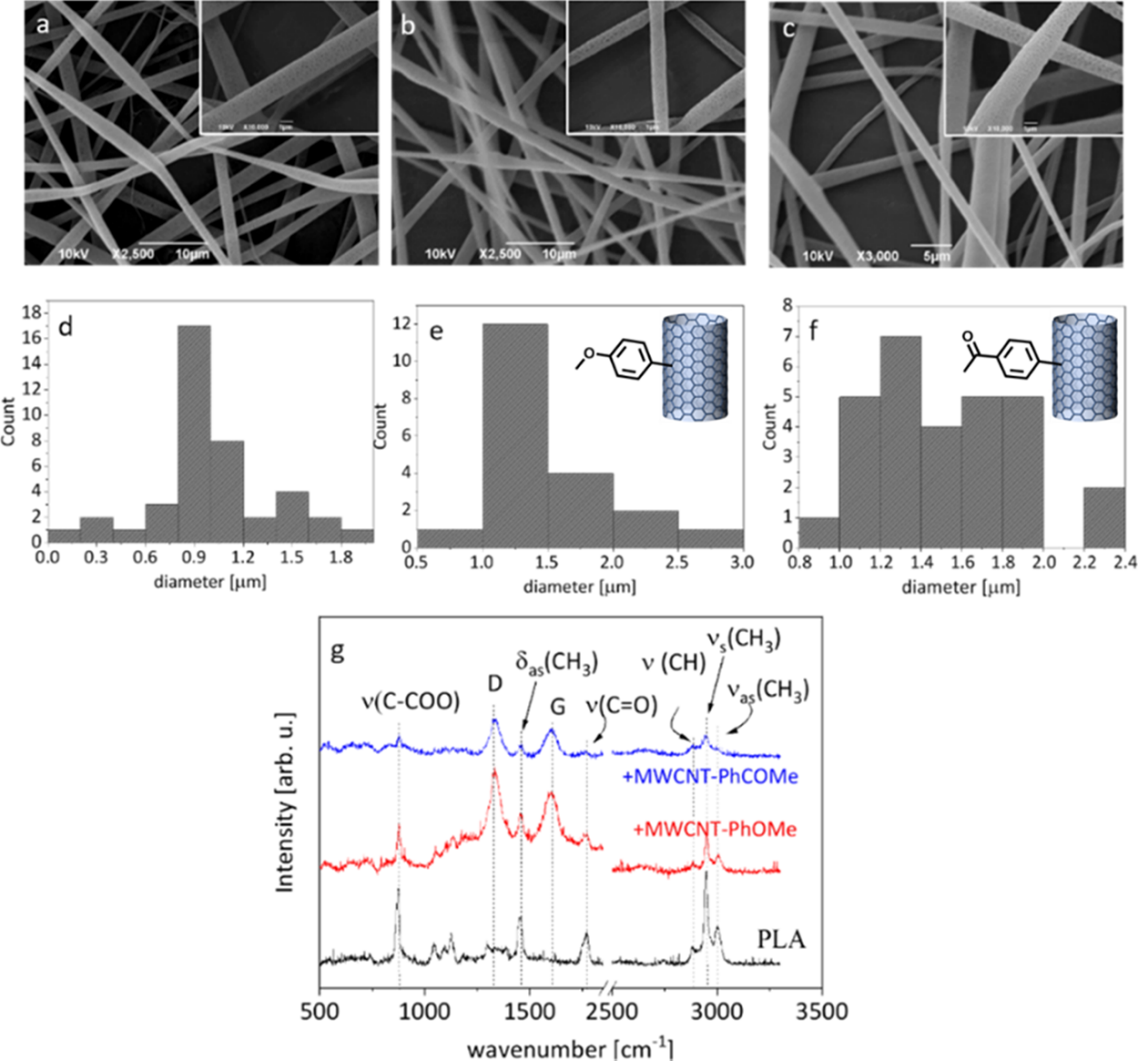
SEM images and corresponding size distributions for (a,d) PLA, (b,e) PLA/MWCNT-PhOMe, and (c,f) PLA/MWCNT-PhCOMe electrospun fibers. (g) μRaman spectra of the PLA and the PLA/MWCNT fiber mats.

Composite fibers were fabricated using PLA as a polymer matrix, either pure or with 1 wt % MWCNTs of either MWCNT-PhOMe or MWCNT-PhCOMe. The mats were composed of uniform and bead-free fibers, as depicted in SEM (Figure 1a–c), confirming that the organic functionalization allows the inclusion of MWCNTs in the matrix without disturbing electrospinning. A higher SEM magnification reveals a high porosity at the fiber surface. It has been shown that the porosity of PLA electrospun fibers, emerging when PLA is dissolved in a binary solvent system (in the present case chloroform:DMF), arises from the solvent miscibility or its interaction with the water droplets present in the atmosphere.^42^ The average diameter of pure PLA fibers was ∼0.9 μm (Figure 1d). The addition of 1 wt % MWCNT-PhOMe led to a small increase of the fiber diameter to 1.25 μm (Figure 1e). Interestingly, the addition of 1 wt % of MWCNT-PhCOMe resulted in less homogeneous fibers, with diameters ranging from 1 to 2 μm (Figure 1f). The wetting properties of all mats were very similar, as seen in Figure S2 (SI).

Raman spectrometry showed the typical D, G, and 2D peaks of the MWCNTs, as well as the main vibrational peaks of the PLA matrix (Figure 1g). More specifically, the strong vibrational mode at 873 cm^–1^ is assigned to the C–COO stretching, at 1450 cm^–1^ to the CH_3_ asymmetric deformation, and at 1765 cm^–1^ to the C=O stretching, while the lines in the high-frequency region 2880–3000 cm^–1^ are assigned to CH and CH_3_ stretching.^43^

**Figure 2:**
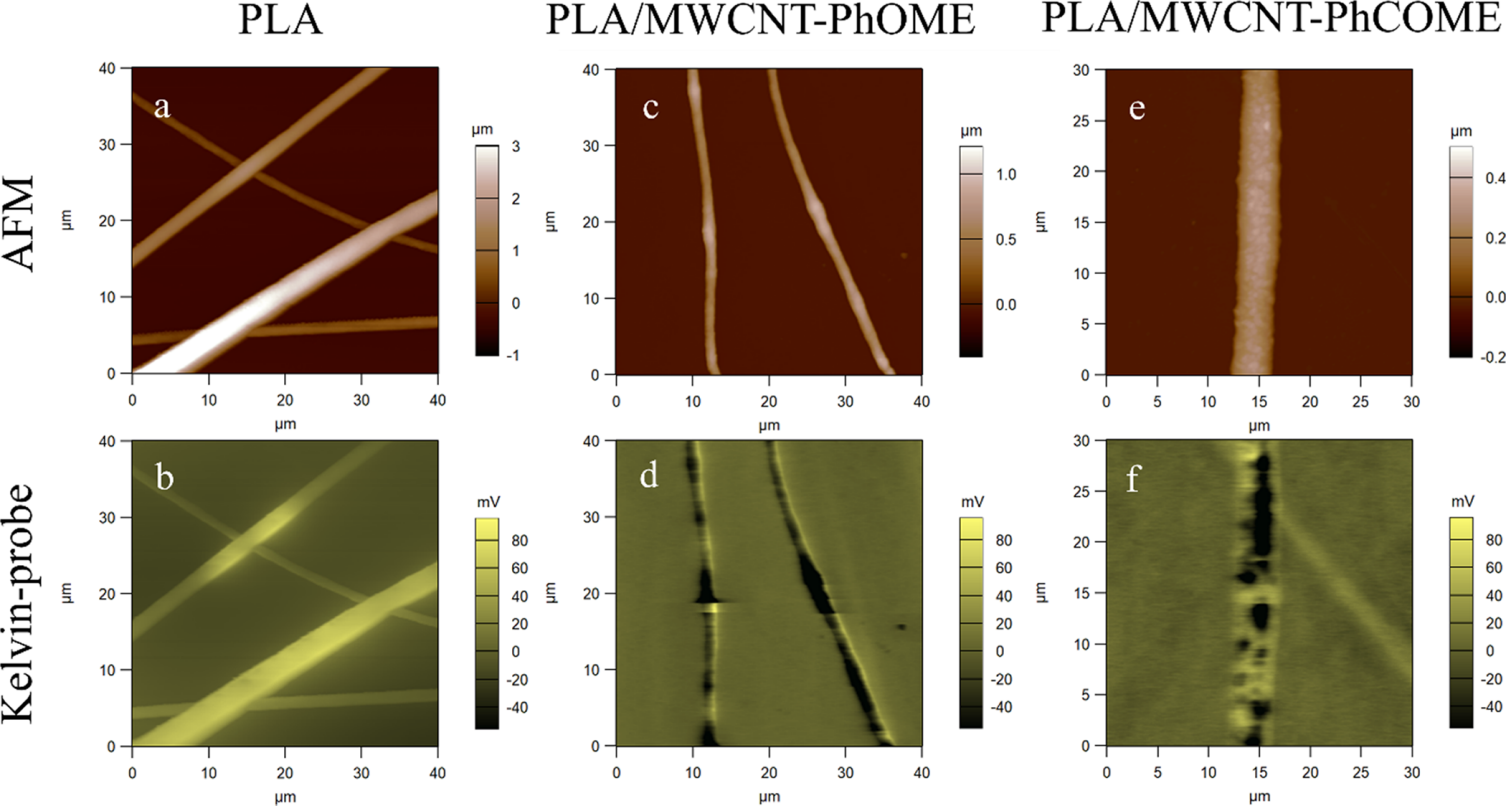
Representative AFM and corresponding Kelvin probe images of the (a,b) PLA, (c,d) PLA/MWCNT-PhOMe, and (e,f) PLA/MWCNT-PhCOMe fiber mats.

AFM and Kelvin probe measurements of the pure PLA scaffolds showed fibers with homogeneous appearance, from both morphological and electrostatic points of view, with the electrical surface potential being almost uniform along the fibers (Figure 2a,b). However, when MWCNTs were embedded into the fibers, an inhomogeneous surface potential was depicted along the fibers, as shown in Figure 2c–f. The surface potential map along the PLA/MWCNT composite fibers consists of various light and dark regions, corresponding to the polymer matrix and the conductive fillers, respectively. The surface potential difference along the PLA/MWCNT-PhOMe fibers has an average value of ∼150 mV. In the case of the PhCOMe functionalization, while the average surface potential difference is ∼150 mV, i.e., similar to the PhOMe one, an uneven distribution of the surface potential was measured, with some areas showing as high as 500 mV (data not shown). Hence, the inclusion of the electrically conductive MWCTs in the insulating PLA matrix induces local variations in the charge distribution at the surface of the fibers.

**Figure 3:**
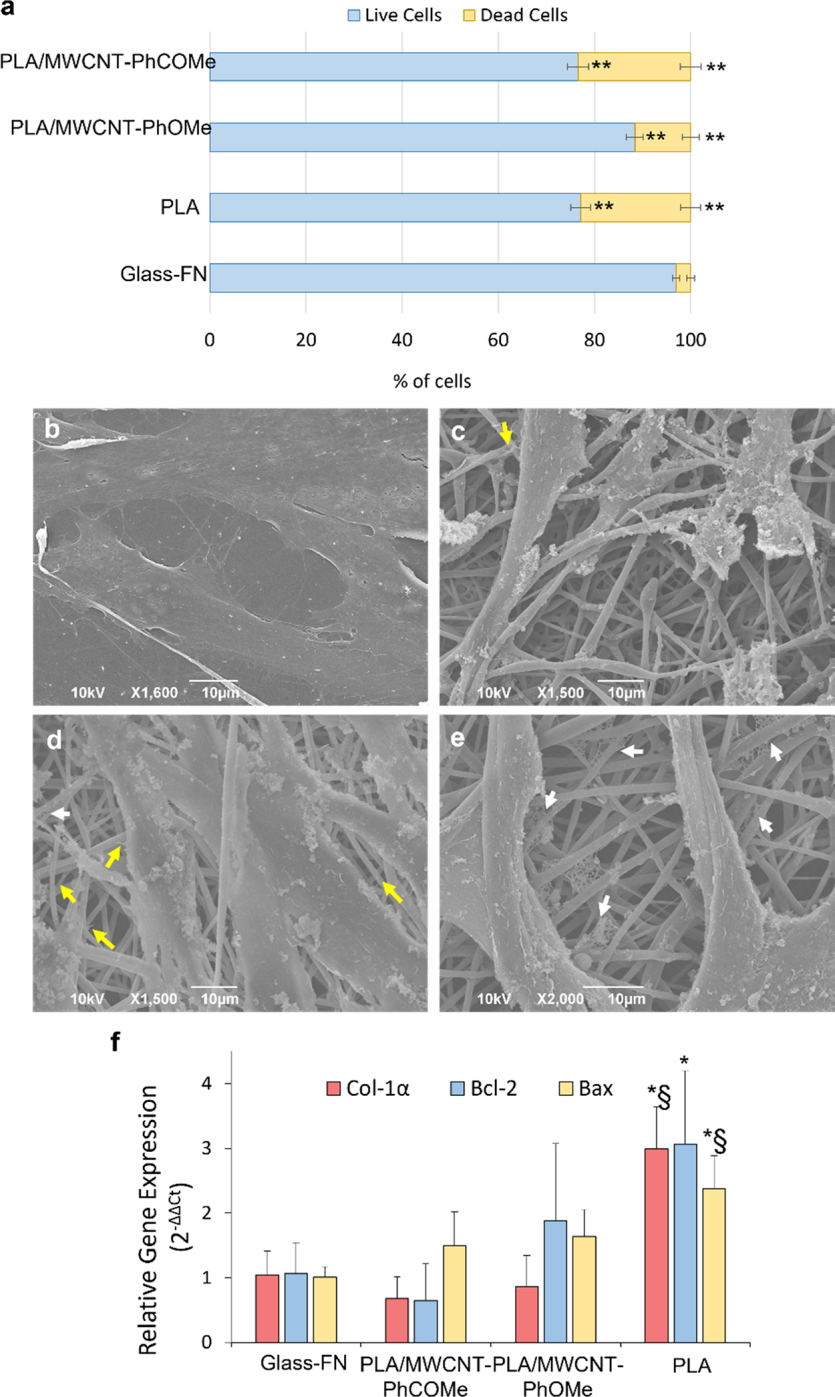
Effect of the fibrous matrices onto primary fibroblasts' morphology and bioactivity. (a) Live/dead staining assay performed on HDFa cells at DIV3, highlighting the % of live and dead cells. Results are reported as mean ± standard error. A Student’s *t*-test, assuming unequal variances, was carried out, considering a value *p* < 0.01 as significant; symbol (**) refers to the significance of difference of all the fibrous matrices with respect to the control glass sample, for both the live and dead cell count ensembles. Glass-FN vs PLA fibers *p* = 5.43 × 10^–10^, Glass-FN vs PLA/MWCNT-PhOMe fibers *p* = 4.67 × 10^–5^, Glass-FN vs PLA/MWCNT-PhCOMe fibers *p* = 6.99 × 10^–9^; PLA fibers PLA/MWCNT-PhOMe fibers *p* = 1.2 × 10^–4^, PLA/MWCNT-PhOMe fibers vs PLA/MWCNT-PhCOMe fibers *p* = 1.4 × 10^–4^. (b–e) SEM micrographs depicting the morphology of HDFa cells at DIV3 onto (b) glass coverslips coated with fibronectin (FN) and (c) matrices of PLA, (d) PLA/MWCNT-PhOMe, and (e) PLA/MWCNT-PhCOMe. Yellow arrows point to the filopodia protruding from adherent cells, while white arrows indicate the presence of secreted extracellular matrix networks. (f) Quantitative results of the PCR analysis for Col-1α, Bcl-2, and Bax gene expression, at DIV3. One-way ANOVA, Scheffé test, *p* < 0.05 significant (mean values ± s.d., *n* = 4; Col-1α: Glass-FN vs PLA fibers *p* = 0.008, PLA/MWCNT-PhCOMe fibers vs PLA fibers *p* = 0.003, PLA/MWCNT-PhOMe fibers vs PLA fibers *p* = 0.004; Bcl-2: Glass-FN vs PLA fibers *p* = 0.023; Bax: PLA/MWCNT-PhCOMe fibers vs PLA fibers *p* = 0.035, Glass vs PLA fibers *p* = 0.029). Symbol (*) refers to the significance of the PLA fiber sample with respect to the control Glass-FN sample. Symbol (§) refers to the significance of the PLA fiber sample with respect to the PLA/MWCNT fiber samples.

Primary adult human dermal fibroblasts (HDFa cells) have been used as a cellular model to observe the effect of our polymeric substrates on cell biocompatibility, morphology, and biofunctionality. Extracts of the fibers were tested (see Experimental Methods) in order to assess whether any harmful components would leak out from the electrospun matrices into the cell culture medium. Preliminary results from MTS assays, performed at early time points, did not highlight any cytotoxicity (Figure S3a, SI) and the cell morphology was comparable to the controls (Figure S3b–e, SI). Next, we further tested the biocompatibility of our scaffolds, with live/dead staining at 3 days of culture (DIV3). Cells plated onto fibronectin (FN)-coated coverslips were considered as a control, given that FN provides optimal conditions for adhesion and growth of skin fibroblasts. In parallel, we analyzed fibroblasts plated on pure PLA and PLA/MWCNT scaffolds. As shown in Figure 3a, all samples under study presented a percentage of live cells above 75%, indicating good substrate biocompatibility. Interestingly, the PLA/MWCNT-PhOMe fibers presented the highest number of live cells (88.35% ± 1.71%), while the PLA/MWCNT-PhCOMe and the pure PLA matrices presented slightly reduced live cell populations (76.56% ± 2.21% and 77.12% ± 2.07%, respectively). Comparable results were also found at DIV1 (Figure S4, SI). The quantitative analysis of the cell area and circularity index as a proxy for the cell morphology (Figure S5) further strengthened the evidence that PLA/MWCNT-PhOMe fibers are a favorable substrate for fibroblasts.

A detailed observation under SEM revealed the presence of spread-out, adherent fibroblasts with several filopodia protruding from the cell membrane for all scaffolds (Figure 3b–e, yellow arrows). Notably, as seen in Figure 3c, the surface of the cells in contact with the plain PLA fibers is rougher with numerous extracellular deposits on their outer membrane, indicating a cellular effort to adapt to a less favorable environment. Interestingly, all of the samples grown in contact with the 3D fibrous matrices revealed the presence of several small networks (Figure 3b–e, white arrows), likely due to newly secreted ECM proteins, although SEM imaging does not allow precise quantification of ECM networks.

The effect of the substrates in regulating cell proliferation and enhancing ECM synthesis was investigated by RT-qPCR. The expression of B-cell lymphoma 2 (Bcl-2) and associated X protein (Bax), together with collagen type I (Col-1α) genes, were evaluated on fibroblasts grown onto the 3D fibrous matrices at DIV3. Cells cultured on glass slides were considered as control. Bcl-2 and Bax belong to the family of genes involved in the regulation of apoptotic cell death. Bcl-2 is an antiapoptotic gene, whose expression is correlated to that of the Bax gene that describes the cells’ apoptotic activity. In recent years, both genes have been used as markers to analyze the degree of cellular apoptosis.^44^ Col-1α is closely related to the ECM formation and deposition, specialized functions of fibroblasts^45,46^ Overall, these three genes constitute reliable markers for the physiological fibroblast proliferation, adhesion, and migration, as Bcl-2 and Bax control cellular apoptosis, while Col-1A controls the extracellular matrix formation, thus promoting physicochemical interactions and communication among the cells and the fibrous scaffold.

The RT-qPCR results of cells grown onto plain PLA substrates revealed a statistically significant increase of all gene expression, compared to the control sample grown on glass (Figure 3f). In the case of composite PLA/MWCNT-PhOMe or PLA/MWCNT-PhCOMe scaffolds, despite some fluctuations, the mRNA expression levels of Bcl-2 and Bax did not show statistically significant differences with respect to the control samples. This suggests that PLA/MWCNT-PhOMe or PLA/MWCNT-PhCOMe fibers do not affect mitotic/apoptotic activity. On the contrary, a significant increase of Bcl-2 and Bax in cells grown on pure PLA scaffolds evidenced that the polymer affects cell homeostasis (Figure 3f).

As already mentioned, collagen forms an integral part of the ECM, providing structural support to the tissues and the anchorage frame for growing cells. In this study, fibroblasts cultured on plain PLA fibers increased their mRNA collagen expression 3-fold. On the other hand, the presence of MWCNT-PhOMe or MWCNT-PhCOMe fillers in the PLA matrix modulated the collagen production to values comparable to the control sample (Figure 3f), thus mitigating the cellular stimuli mediated by the PLA polymer.

**Figure 4:**
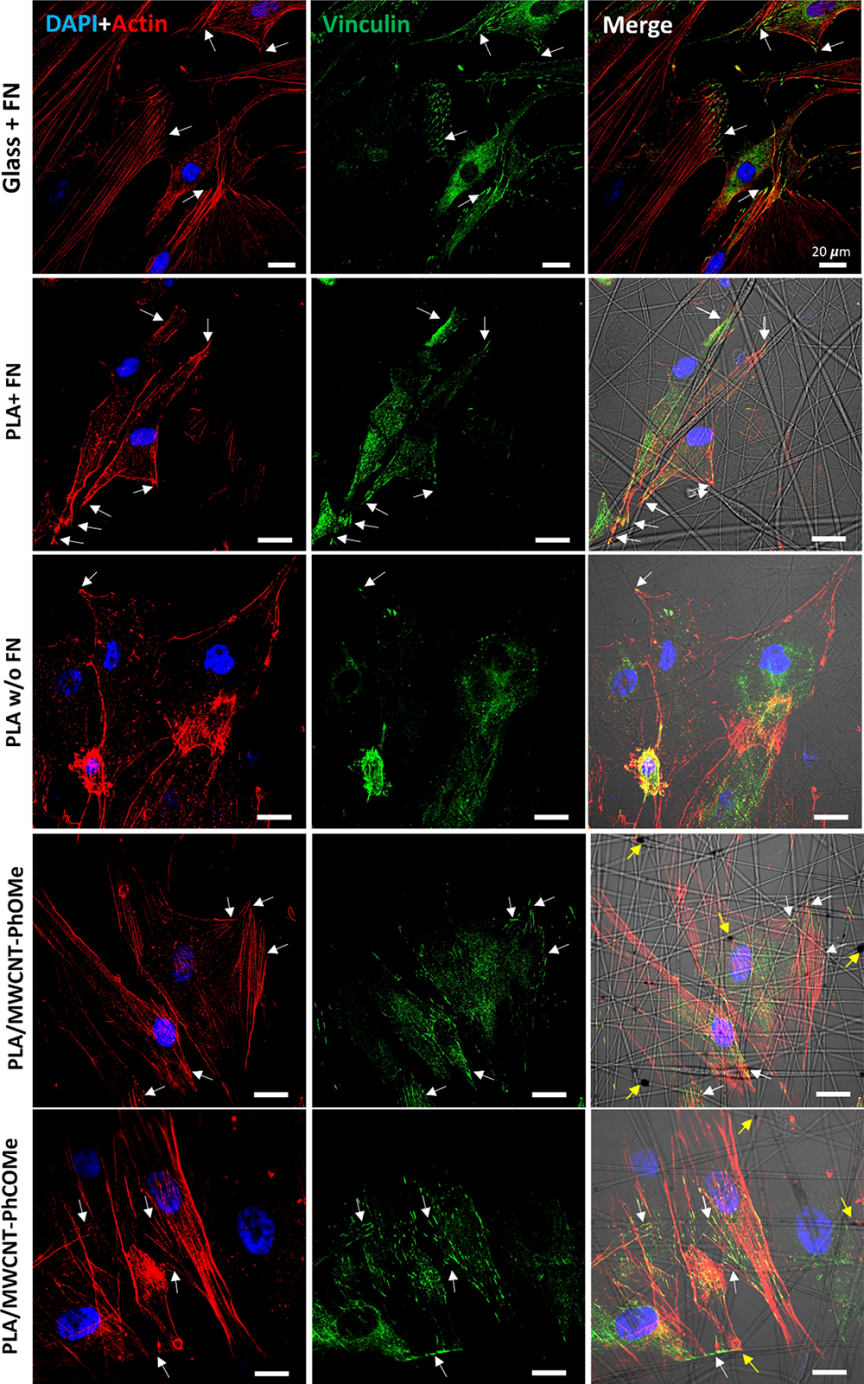
Effect of the fibrous matrices onto the fibroblasts' focal adhesion complex arrangements: confocal images of HDFa cells plated onto different fibrous samples or the control, glass coverslips at DIV3. Cell nuclei are stained with DAPI (blue), while the cytoskeletal actin fibers are stained red (Alexa Fluor 546 Phalloidin). Green shows vinculin. White arrows in the images indicate the focal adhesion complex (colocalization of green and red signals). Yellow arrows in the merged images indicate the MWCNTs encapsulated into the polymeric fibers. Scale bar 20 μm.

In a third set of experiments, we investigated the interaction of the primary adult fibroblasts with the different substrates in terms of their morphology, adhesion, and spatial arrangements. Integrins are trans-membrane proteins, which, by linking the actin fibers of the cytoskeleton with the outer cellular milieu, play a critical role as mechano-sensory receptors in dermal fibroblasts, ultimately regulating tissue homeostasis and skin wound healing.^47^ A stable cell adhesion is reached through the formation of a focal adhesion (FA) point, which comprises a plethora of intracellularly associated protein complexes, such as vinculin, talin, α-actinin, paxillin, tensin, zyxin, and focal adhesion kinases.^48^ Vinculin is generally considered one of the universal markers for the adhesion complex formation. In order to study the influence of the MWCNTs-loaded matrices on fibroblasts structure and function, we analyzed the expression of actin fibers and vinculin in fibroblasts seeded onto different scaffolds by confocal imaging. As expected, HDFa cells plated onto FN-coated coverslip (control sample) showed a spread morphology, bundles of actin filaments, and a diffused vinculin staining indicating a uniform cell adhesion to the glass substrate and adequate functional activity of the fibroblasts (Figure 4, first row). On the contrary, cells grown on pure PLA fibers revealed more disorganized actin filaments, and their vinculin staining appeared clumped around the cell nuclei, suggesting difficulty in adhering onto the bare polymer scaffold (Figure 4, third row). Therefore, to ensure the actual influence of the scaffolds onto fibroblast adhesion, we decided to include FN-coated PLA fibers in the confocal investigation. In this case, HDFa cells showed a spread morphology, well-defined actin filaments, and vinculin staining visible as short green hyphens, indicating that focal adhesion complexes formed preferentially towards the edges of the cells, where they could anchor to the composite matrices (Figure 4, second row). We next analyzed cells plated onto the PLA/MWCNT scaffolds. On both PLA/MWCNT-PhCOMe and PLA/MWCNT-PhOMe fibers, fibroblasts exhibited a spread morphology, with stretched actin bundles and sharp vinculin clusters (visible as green spots or short traits in Figure 4), even without resorting to the extra adhesion protein coating (see also Figures S6 and S7). Remarkably, the colocalization of the vinculin and actin immunoreactivities (Figure 4, white arrows), indicating specific focal adhesion points, was more pronounced at sites where the MWCNTs were in the proximity of the cell (Figure 4, fourth row, yellow arrows).

A quantitative analysis of the vinculin traits within the cells in contact with various substrates and their colocalization with either actin filaments or electrospun fibers was performed (Figure S7). The presence of MWCNTs within the polymeric matrices led to an increase in the number of vinculin traits per cell. This is in accordance with the morphological assessment presented above, where cells with a wider area (as observed onto the PLA/MWCNT-PhOMe fibers) correlate with a higher number of vinculin traits, hence a stronger adhesion. In parallel, the average vinculin length in PLA/MWCNT-PhOMe fibers was comparable to the control glass samples, excluding the possibility of longer vinculin traits as an additional mechanism to promote cell adhesion. Remarkably, these vinculin traits colocalize with actin filaments, thus constituting functional focal adhesion complexes, and preferentially aggregate in correspondence with the polymeric network (73,34 ± 3,79%, 56,88 ± 3,73%, 47,14 ± 4,66%, for the PLA/MWCNT-PhOMe fibers, the PLA/MWCNT-PhCOMe fibers, and the PLA fibers, respectively) (Figure S7).

Those results suggest the possibility that the appropriate growth and development of fibroblasts on PLA/MWCNTs is due to the establishment of functional adhesion complexes, favored by the local electric fields generated by the MWCNTs. As such, a perturbed extracellular local environment can tune the transmembrane protein arrangements.^49^

**Figure 5:**
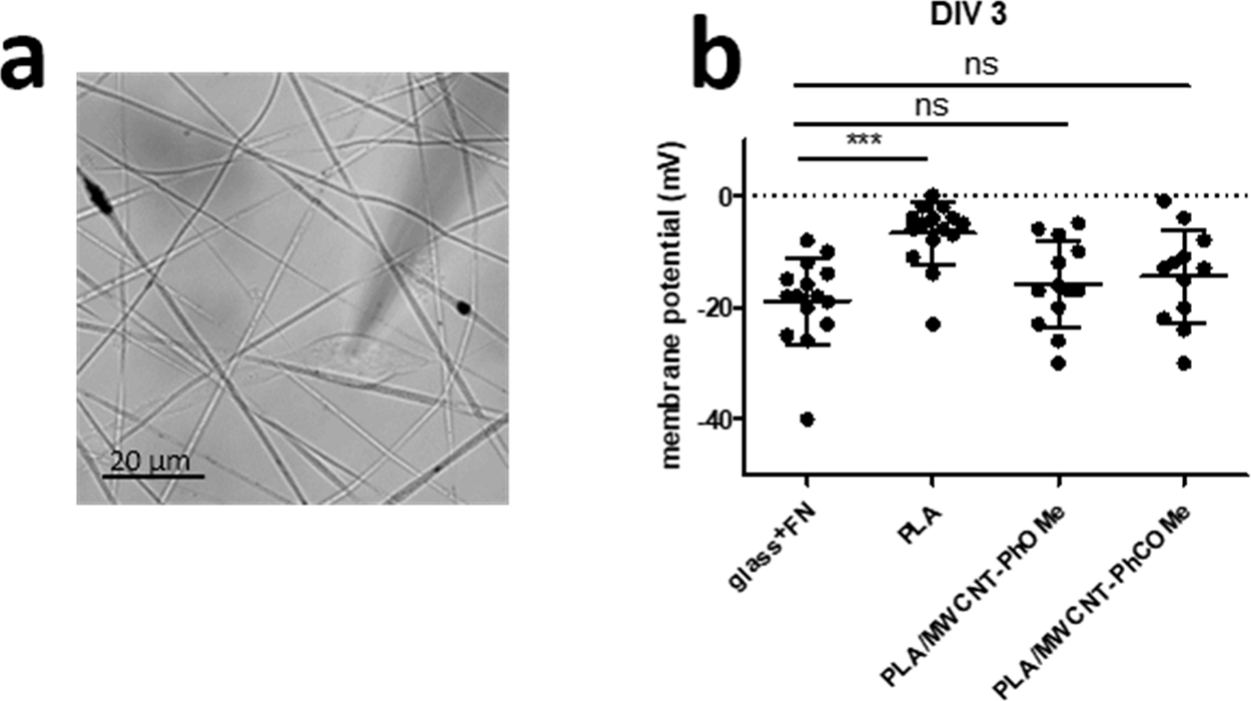
Effect of fibrous substrate composition on fibroblast membrane potential. (a) DIC image of a cultured HDFa cell showing a patched fibroblast during an electrophysiological recording. Scale bar 20 μm. (b) Membrane potential (mV) measurements recorded from HDFa cells at DIV3 on various substrates (*n* = 13–15 cells per condition, from five different preparations). *V*_mem_ values below 0 are considered hyperpolarized. ns, nonsignificant; (***) *p* < 0.001; one-way ANOVA followed by Dunnett’s post test.

The effects of the PLA and composite scaffolds on the cell membrane potential, *V*_mem_, were analyzed by the patch clamp technique (Figure 5a). At DIV3, the membrane potential of fibroblasts seeded on FN-coated glass was hyperpolarized (*V*_mem_ = −18.8 ± 1.2 mV, *n* = 15 cells), whereas that of fibroblasts grown on a pure PLA scaffold was closer to zero (*V*_mem_ = −6.7 ± 1.5 mV, *n* = 15 cells, Figure 5b). Interestingly, upon the inclusion of the MWCNTs in the PLA matrix, the fibroblast *V*_mem_ was more hyperpolarized than the pure PLA and comparable to that measured for the control cells seeded on FN-coated glass. More specifically, we measured an average *V*_mem_ = −16.14 ± 2.00 mV and −13.69 ± 2.34 mV from fibroblasts seeded on PLA/MWCNT-PhOMe and PLA/MWCNT-PhCOMe, respectively (Figure 5b). A similar analysis conducted on the same samples at an earlier time point (Figure S9, SI) confirmed that control fibroblasts on FN-coated glass exhibited hyperpolarized *V*_mem_ already at DIV1 (*V*_mem_ = −18.11 ± 2.94 mV), suggesting that hyperpolarized V_mem_ correlates with favorable growing conditions. In contrast, pure PLA fibers promoted a significant variation of fibroblast *V*_mem_ to values close to zero (*V*_mem_ = −4.53 ± 1.19 mV). In line with the idea that the polarization fields, generated by the inclusion of MWCNTs in the insulting PLA matrix, promote the hyperpolarization of the fibroblasts, we quantified that on PLA/MWCNT-PhOMe and PLA/MWCNT-PhCOMe fibroblasts *V*_mem_ values were similar to control also at DIV1 (*V*_mem_ = −13.81 ± 2.78 and −12.77 ± 2.38 mV from fibroblasts seeded on PLA/MWCNT-PhOMe and PLA/MWCNT-PhCOMe, respectively).

Those results indicate that after seeding, the presence of MWCNTs in the PLA fibers reverts the effects of pure PLA on fibroblasts *V*_mem_ already at DIV1, by restoring it to hyperpolarized values and preserving it in the following days.

Summarizing the results on the effect of the electrostatic fields interfaced with the fibroblasts, differences are seen in the biofunctions of fibroblasts grown in the presence or absence of these fields. Cells grown on PLA/MWCNT-PhOMe and PLA/MWCNT-PhCOMe, in which the embedded MWCNTs generate electrostatic fields (see Figure 2), present an overall healthier state, both from a morphological point of view and from a protein expression level one. Morphologically, cells are well-spread on all substrates, but when on pure PLA their surface appears rougher, as seen in SEM. Such roughness, which is likely caused by the presence of ECM vesicles at the surface of the cells, is indicative of “stress” related to difficulties in adapting to the environment,^50^ even though pure PLA samples are biocompatible with a high percentage of viable cells. This interpretation is corroborated by the increased collagen expression, which has been attributed to a response to inflammation caused by PLA.^51−54^ The evidence that pure PLA provides an unfavorable environment for fibroblasts is further supported by immunofluorescence microscopy data showing the uneven distribution and clumping of vinculin and actin proteins inside the cells (Figure 4) as a proxy for the cells’ difficulty to adhere and spread. Furthermore, fibroblasts grown on pure PLA show a compromised mitotic/apoptotic activity. On the contrary, the overall morphology and substrate adhesion of the fibroblasts interfaced with the electrostatic fields generated by MWCNTs are similar to the control samples and are considered physiological. Similarly, mitotic/apoptotic activity and collagen expression are also restored to the values observed in the control sample and thus considered physiological. From a bioelectrical point of view, only fibroblasts interfaced with electrostatic fields exhibit physiological *V*_mem_ values. This important result is in line with the central idea of the current research on how interfacing cells with an appropriate electrical environment can shape the bioelectric cell functions.

**Figure 6:**
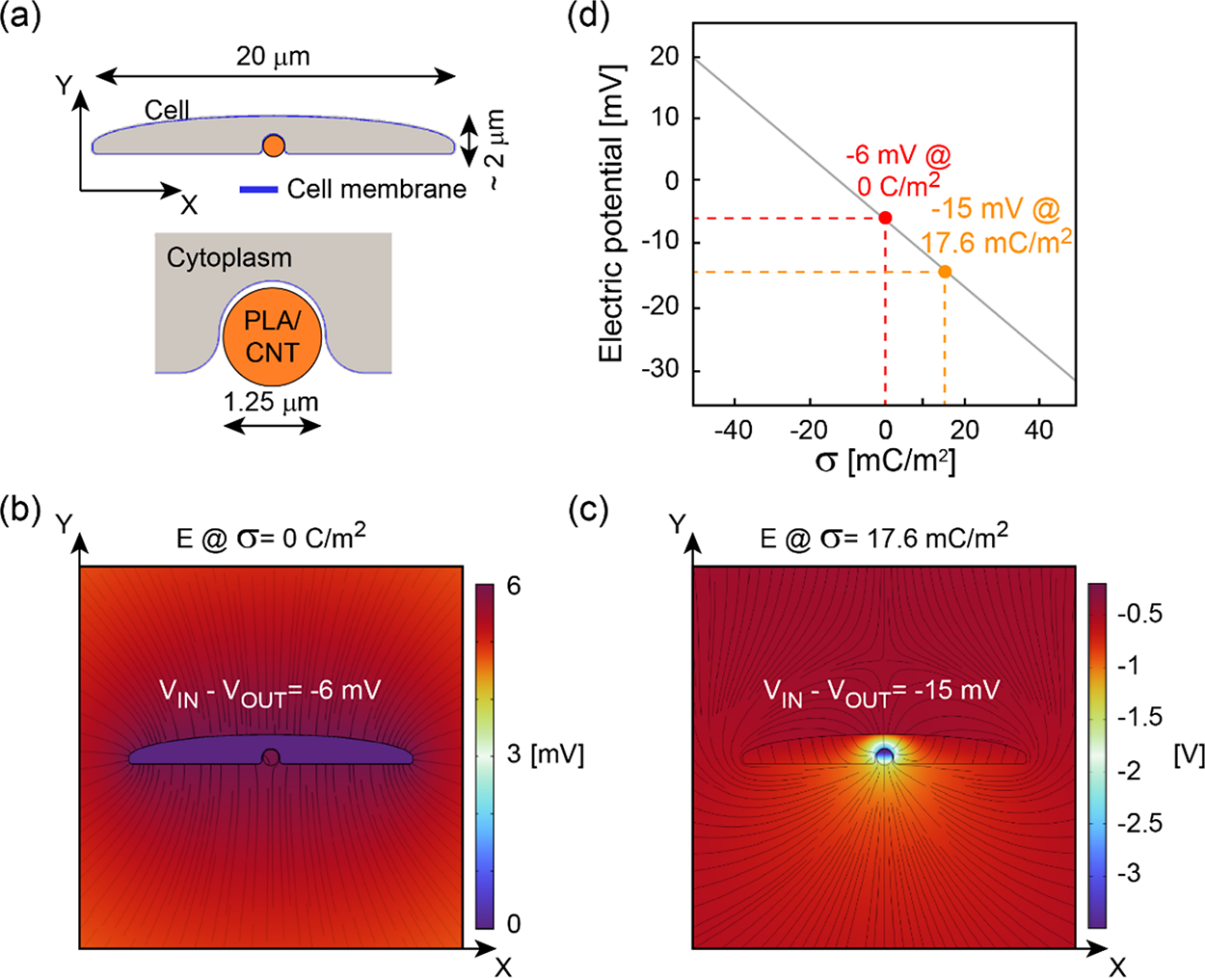
Numerical simulations of the substrate-related induced electric field. (a) Schematic of the simulation. (b,c) Electric field calculated inside and outside the cell when PLA (b) and PLA/MWCNTs (c) were considered. The corresponding membrane potential is also reported. (d) Dependence of the membrane potential on the electric charge deposited on a PLA-like fiber. For all simulations, convergence testing was performed in order to guarantee the accuracy of the results.

In order to deepen our understanding on how the interfacial electric field affects the membrane potential, we have conducted numerical simulations (Figure 6), where two situations were considered: (i) cells grown on PLA and (ii) cells grown on PLA/MWCNTs.

Figure 6a depicts the schematics of the numerical simulations. Here, a single cell is considered in contact with a single fiber. The cell cytoplasm has permittivity ε = 80, while the membrane has ε = 2.5 and thickness equal to 10 nm. The surrounding medium is an electrolyte with ε = −10^4^ (ideal conductor). For simplicity, given the minimum permittivity difference between PLA (4.5) and PLA/MWCNTs composite (∼3), a value equal to 4.5 (PLA) was adopted for the fiber in all simulations (this does not affect the overall prediction of the simulations). Our initial assumption was to consider the cell membrane potential in resting conditions for a value of *V*_IN_ – *V*_OUT_ = −6 mV (as from the experimental data, Figure 5b). This situation was imposed both for an isolated cell and for a cell in contact with an electrically neutral PLA fiber (Figure 6b). Differently, when the PLA/MWCNTs composite was considered, the experimental data (Figure 5b) showed a membrane potential close to *V*_IN_ – *V*_OUT_ = −15 mV. We have then numerically investigated the physical mechanism leading to such a change in terms of electric charges/electric field, with the results shown in Figure 6c,d. Here, it is found that adding positive charges to the electrically neutral PLA fiber underneath the cell leads to a shift of *V*_mem_ towards more negative values, a process known as hyperpolarization. In particular, the charge density (σ) determining a shift from −6 to −15 mV is found to be equal to 17.6 mC/m^2^. The positive sign of the charges is consistent with our experimental evaluations that see the PLA/MWCNT fibers positively charged, as explained in our previous publication.^32^ Importantly, the value of −15 mV is calculated as the difference between the internal potential of the whole cell (*V*_IN_), which we evaluated by averaging the potential within the whole cell volume, and the cell external potential (*V*_OUT_), computed as the average of the electrostatic potential along the entire outer boundary of the cell membrane. In this way, our numerical estimation is reasonably close to the outcome of the measurements based on the patch-clamp technique.

Our working hypothesis is that MWCNT fillers provide an electrical milieu that allows, or even prompts, the hyperpolarization of *V*_mem_ to the physiological value, while the absence of a suitable electrical milieu prevents the establishment of a hyperpolarized *V*_mem_. Interestingly, the average *V*_mem_ of fibroblasts grown on PLA/MWCNT-PhOMe was more negative than that measured in PLA/MWCNT-PhCOMe. This difference may be ascribed to their surface potential data (Kelvin probe measurements, Figure 2) and, ultimately, to their diverse dispersion within the fibrous polymeric network. These results are in accordance with our previous results on primary neurons grown on P(3HB), a biocompatible biopolymer where cells retained their physiological electrophysiology properties, including *V*_mem_, only when graphene nanoplatelets were embedded in the P(3HB) matrix.^14^ The present study reports measurements of *V*_mem_ of human dermal fibroblasts plated onto biocompatible electrospun matrices. We reveal that the presence of electrically conductive MWCNTs in the PLA matrix makes the whole system a more suitable environment for the morphological and functional development of fibroblasts by preserving the physiological *V*_mem_ values of these nonexcitable cells.

Bioactive scaffolds provide an electrically suitable environment for the attachment, growth, and spreading of the fibroblasts. The preservation of the physiological state of the membrane potential is essential for the correct biofunctionality of the cells, and it becomes possible only when a suitable electrical milieu is provided.

## Conclusions

We have investigated the effect of embedding electrically conductive MWCNT in a PLA matrix on human dermal fibroblast cultures. MWCNTs were covalently functionalized with two different functionalities, namely, *p*-methoxyphenyl (PhOME) and *p*-acetylphenyl (PhCOMe). The covalent functionalization approach allowed us to obtain homogeneous electrospun nanofibers while retaining the morphological and electrical properties of the MWCNTs. The inclusion of electrically conductive MWCNTs in the PLA matrix resulted in a spatially heterogeneous surface electric potential on the scaffolds. Characterization of the cellular system under study, for short-time interaction, revealed that the cells grown on the PLA/MWCNT scaffolds presented a healthy morphology and the ability to establish focal adhesion complexes and produce ECM proteins necessary for their growth and further proliferation. Finally, electrophysiology measurements showed that fibroblasts grown on the PLA/MWCNT scaffolds were able to preserve a physiological value for their *V*_mem_. On the other hand, fibroblasts plated on pure PLA scaffolds presented a slightly shrunk morphology, ECM proteins were overexpressed in comparison to the control sample, focal adhesion complexes appeared disorganized, and cells did not retain a physiological *V*_mem_ value. PLA is considered a well-established scaffold material, and as also observed here, cells in direct contact with it do retain good viability. However, our findings also report that cells do not grow to their full functional capacity when in contact with pure PLA. On the other hand, the introduction of electrically conductive MWCNTs in the polymer matrix locally modified the *V*_mem_ of single cells, resulting in their bioelectrical development. The experimental results are supported by numerical simulations that show that the bioelectrical development of the cell membrane is established only in the presence of the electrostatic fields created by the addition of positive charges at the cell/material interface.

These important results substantiate how a suitable electrical interface can shape the extracellular milieu to regulate the *V*_mem_ of nonexcitable cells, such as fibroblasts, and their subsequent biofunctional development.

## Supporting Information Available

Synthesis scheme and characterization (Raman spectra and TGA data) of the MWCNT-PhOMe and the MWCNT-PhCOMe; water contact angle measurements of the PLA, PLA/MWCNT-PhOMe and PLA/MWCNT-PhCOMe fibers; MTS assay results and confocal imaging at DIV1 and DIV2 of HDFa cells grown in contact with the fibers extract media; live/dead staining assay results at DIV1 of HDFa cells grown in direct contact with the PLA, PLA/MWCNT-PhOMe, and PLA/MWCNT-PhCOMe fibers; cell morphology analysis in terms of average area and average circularity index of HDFa cells plated onto the PLA, PLA/MWCNT-PhOMe, and PLA/MWCNT-PhCOMe fibers; confocal images of HDFa cells plated onto PLA/MWCNT-PhOMe and PLA/MWCNT-PhCOMe fibers at DIV3; confocal images of HDFa cells plated onto PLA/MWCNT-PhOMe+FN and PLA/MWCNT-PhCOMe+FN fibers at DIV3; analysis of the focal adhesion complex in terms of normalized number of vinculin traits, average length of vinculin traits, and vinculin traits colocalization with actin filaments and/or electrospun fibers; membrane potential measurements recorded from HDFa cells at DIV1 onto glass+FN, PLA, PLA/MWCNT-PhOMe, and PLA/MWCNT-PhCOMe fibers; and list of the primer acquired and relative gene sequence description (PDF)
