# Supplementary material for: Modified Carbon Nanotubes Favor Fibroblast Growth by Tuning the Cell Membrane Potential
Source: ACS Appl Mater Interfaces. 2024 Jan 11;16(3):3093–105. doi: 10.1021/acsami.3c14527 (PMC10811621; doi:10.1021/acsami.3c14527)
Supplement: Supplementary file 1 — am3c14527_si_001.pdf [file am3c14527_si_001.pdf]

Supplementary Information

Modified Carbon Nanotubes Favor Fibroblast Growth by Tuning the Cell Membrane Potential

Giulia Suarato [1,i], Samuel Pressi[2,3], Enzo Menna[2,3,], Massimo Ruben[1,ii], Enrica Maria Petrini[1,*], Andrea Barberis[1], Dalila Miele[4], Giuseppina Sandri[4], Marco Salerno[1], Andrea Schirato[1,5,6], Alessandro Alabastri[6], Athanassia Athanassiou[1], Remo Proietti Zaccaria [1,*] and Evie L. Papadopoulou[1,iii,*]*

[1] Istituto Italiano di Tecnologia via Morego 30, 16163 Genova (Italy)

[2] Department of Chemical Sciences, University of Padua, via Marzolo 1, 35131, Padova (Italy)

[3] Interdepartmental Centre Giorgio Levi Cases for Energy Economics and Technology, University of Padua, via Marzolo 9, 35131, Padova (Italy)

[4] Department of Drug Sciences, University of Pavia, via Taramelli 12, 27100 Pavia (Italy)

[5]Dipartimento di Fisica, Politecnico di Milano, P.zza Leonardo da Vinci 32, Milan 20133 (Italy)

[6] Department of Electrical and Computer Engineering, Rice University, 6100 Main Street, Houston, TX, 77005 (USA)

Corresponding Authors e-mails:

Evie L. Papadopoulou (paraskevi.papadopoulou@iit.it); Enzo Menna (enzo.menna@unipd.it); Enrica Maria Petrini (enrica.petrini@iit.it); Remo Proietti Zaccaria (remo.proietti@iit.it).

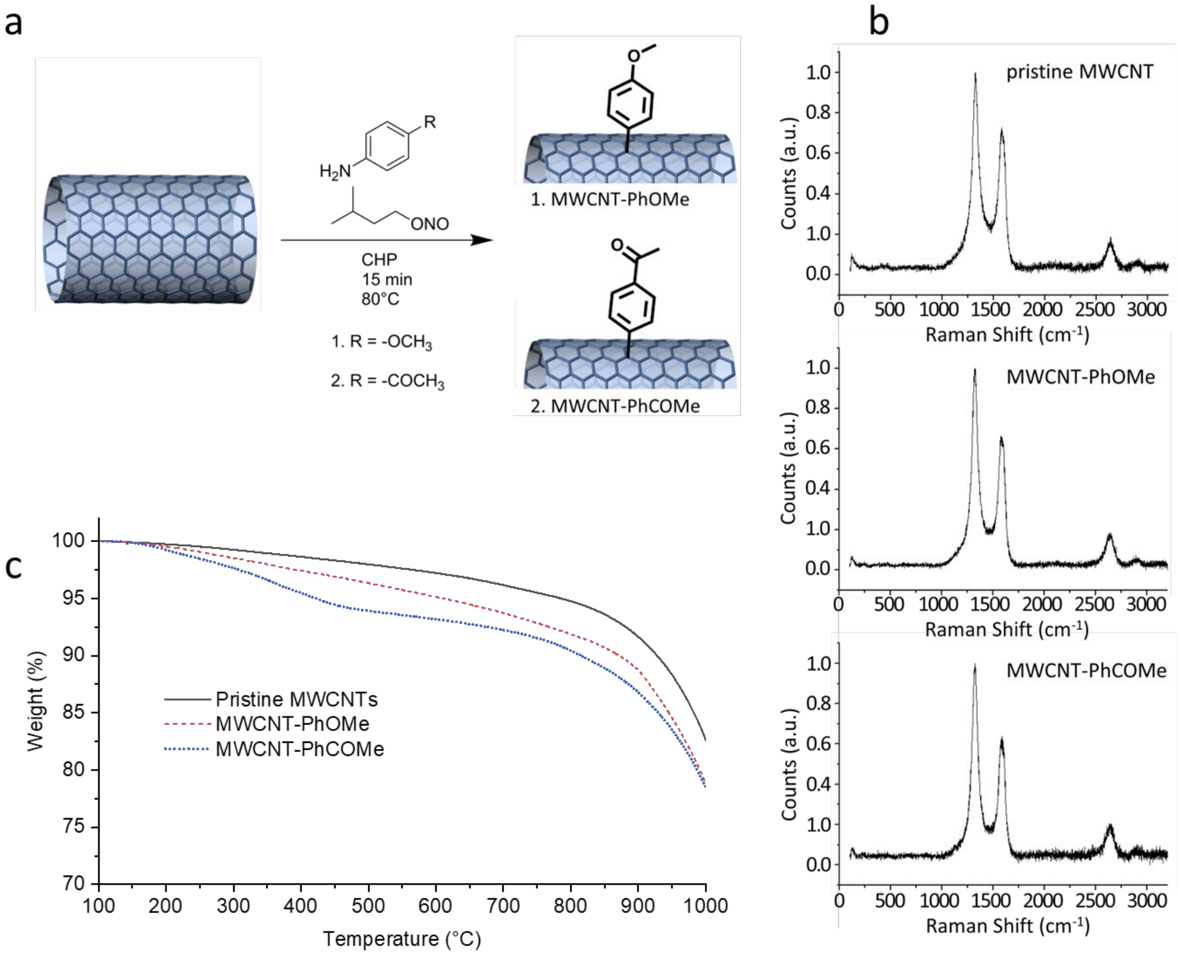

Figure S1: (a) synthesis scheme; (b) Raman Spectroscopy data and (c) TGA data of MWCNT-PhOMe and MWCNT-PhCOMe (CHP = N-Cyclohexyl-2-pyrrolidone).

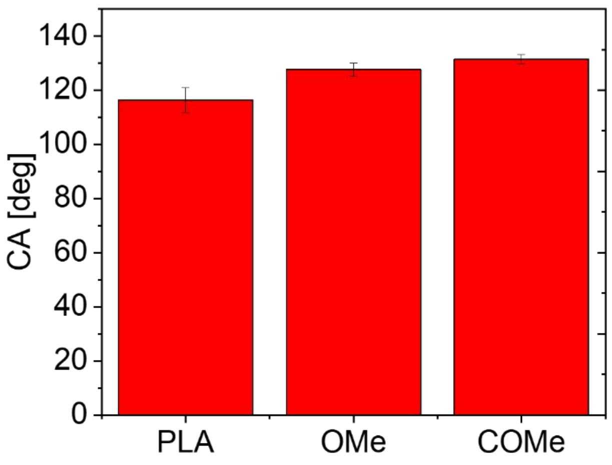

Figure S2: Water contact angle for PLA, PLA/MWCNT-PhOMe and PLA/MWCNT-PhCOMe.

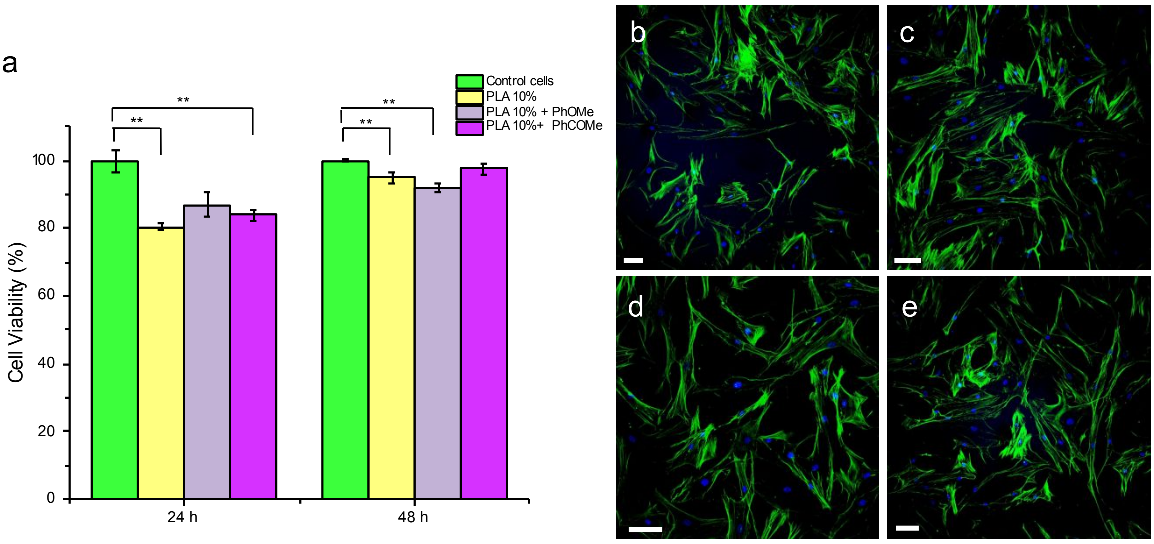

Figure S3: Effect of the fibers extracts onto the HDFa cell viability: (a) MTS assay results at DIV1 and DIV2 in control medium and extract-containing media. A Student's *t*-test, assuming unequal variances, was carried out, considering a value $p<0.01$ (**) as significant. Confocal images at 24 h of culture in the presence of (b) normal growth media; (c) extract obtained from PLA fibers; (d) extract obtained from PLA/MWCNT-PhOMe fibers; (e) extract obtained from PLA/MWCNT-PhCOMe fibers. Actin fibers were stained with AF488 Phalloidin (green channel), while the nuclei were highlighted with DAPI (blue channel). Scale bar 100 μm.

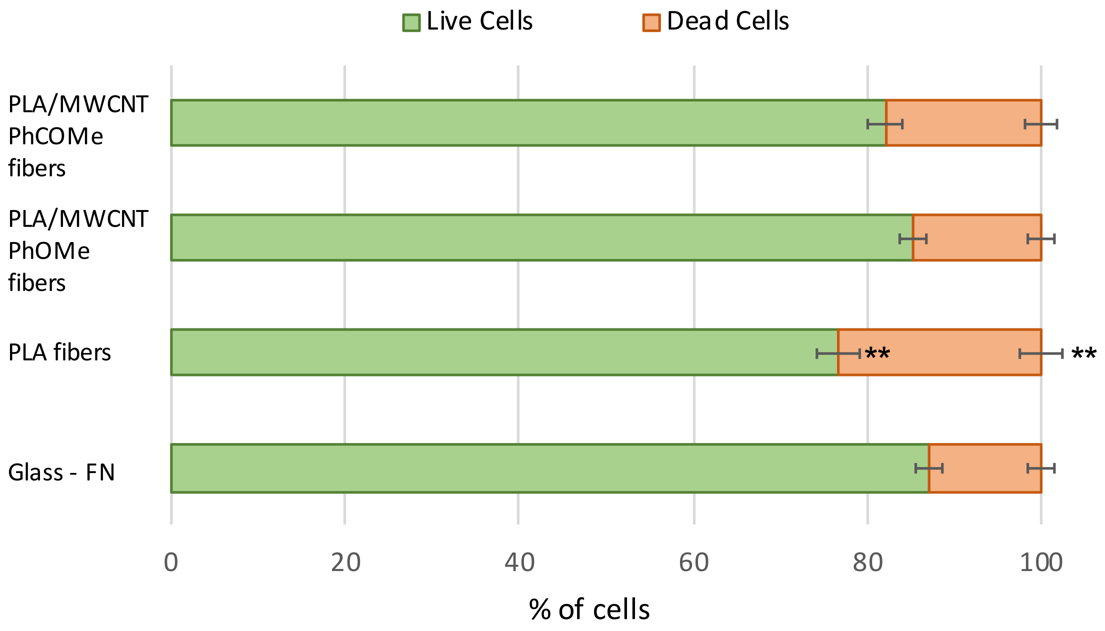

Figure S4: Effect of the fibrous matrices onto the primary fibroblast morphology and bioactivity: Live/dead staining assay performed onto HDFa cells plated for 24 h onto the various samples under study, highlighting the % of live and dead cells. Results are reported as mean ± standard error. A Student's *t*-test, assuming unequal variances, was carried out, considering a value $p<0.01$ as significant; the symbol (**) refers to the significance of the difference of PLA fibers with respect to the control glass sample, for both the live and dead cell count ensembles. Glass vs PLA fibres $p=0.0007$, PLA/MWCNT-PhOMe fibres vs PLA fibres $p=0.004$.

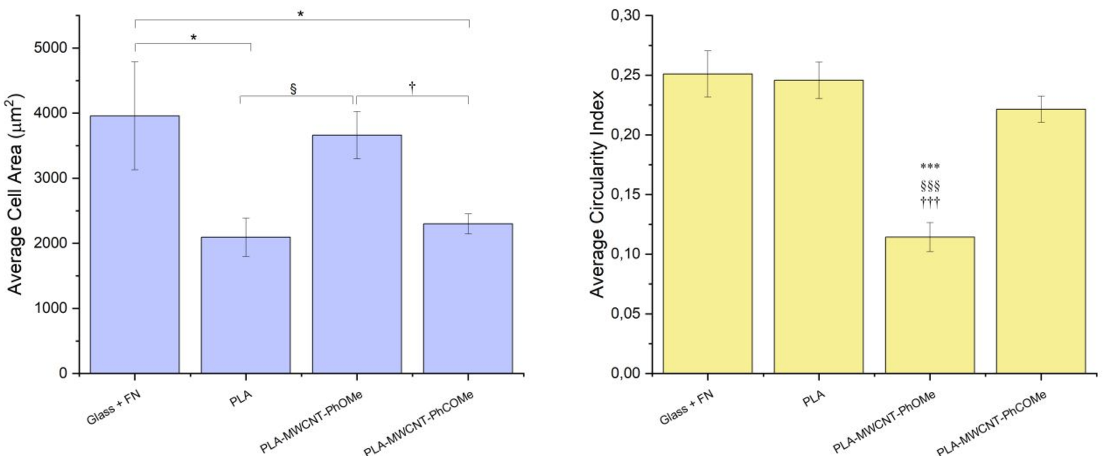

Sample	Average Cell Area (μm^2)	St. err.	Average Circularity Index	St. err.
Glass + FN	3959	828,2	0,251	0,0193
PLA	2093	294,4	0,2458	0,01525
PLA-MWCNT-PhOMe	3661	362,8	0,1143	0,01208
PLA-MWCNT-PhCOMe	2299	153,4	0,2215	0,01103

Figure S5: Fibroblast morphology analysis: Average area (μm^2) and average circularity index of the cells plated onto the various substrates under study. Data are presented as mean value ± standard error. The significance was determined via ONE WAY ANOVA followed by Bonferroni's post-hoc test, considering $p < 0.05$ (*), $p < 0.01$ (**), and $p < 0.001$ (***). More specifically, for the significance, (*) represents the comparison with respect to the Glass+FN sample; (§) highlights the significance of the PLA-PhOMe substrate with the pristine PLA substrate; while (†) pinpoints that the statistical test was performed between the MWCNTs-containing samples.

As noticeable from the reported data, fibroblasts plated onto PLA/MWCNT-PhOMe fibres are the only ones presenting an average area comparable to the control, i.e. grown onto glass coated with the adhesion protein fibronectin, FN. One the other hand, the area of fibroblasts grown on PLA and PLA/MWCNT-PhCOMe substrates was smaller, suggesting an unfavorable adhesion of the fibroblasts onto those fibrous matrices. Considering that fibroblasts preferentially elongate in one direction and extend their thin filopodia and lamellipodia in the surrounding environment, we observed that their circularity indexes, defined as the ratio between the major and minor axes, was [illegible] 0,25. Interestingly, the PLA/MWCNT-PhOMe sample appears to accentuate this morphological feature (C.I. ~ 0,11), coupling the chemical appropriateness with their 3D network fibrous architecture, which has been previously and extensively shown to favor the establishment of elongated cellular phenotypes. Taken together, these quantitative observations further support the identification of the PLA/MWCNT-PhOMe sample as the most suitable substrate for fibroblast cells seeding, adhesion, and growth.

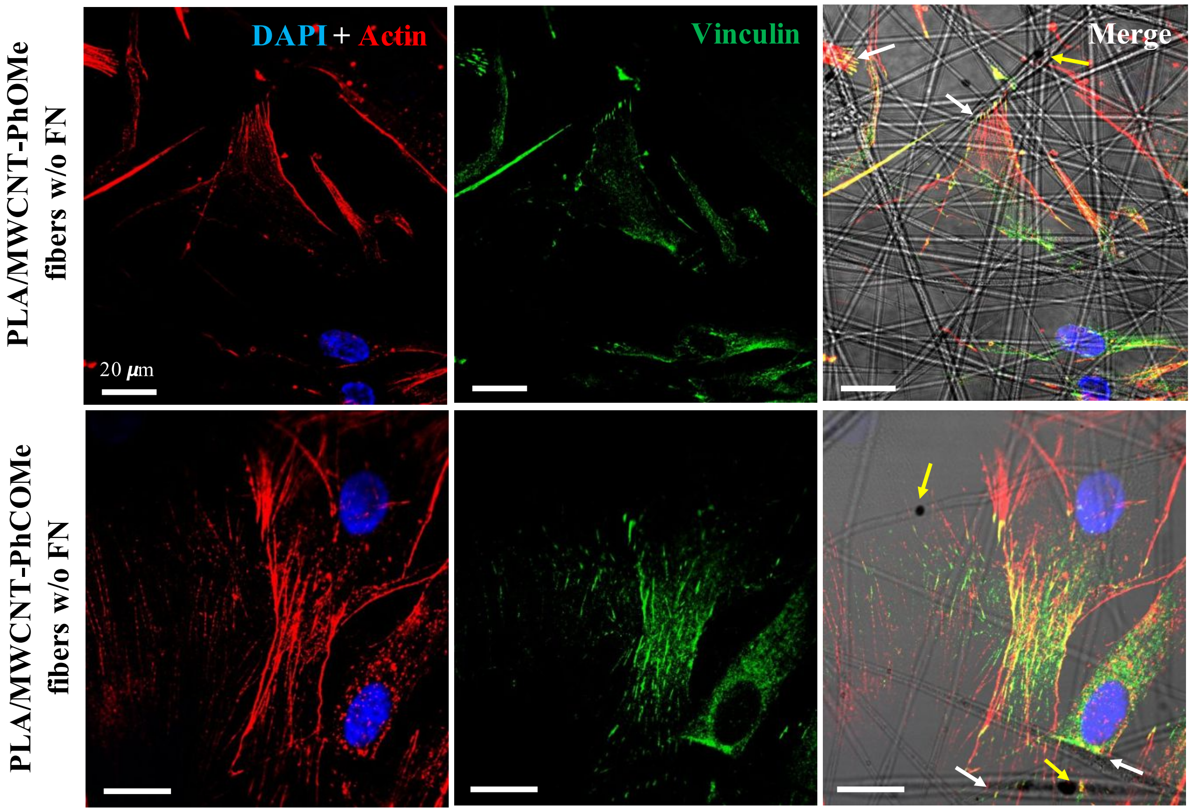

Figure S6: Effect of the fibrous matrices onto the fibroblasts focal adhesion complexes arrangements: confocal images of HDFa cells plated onto PLA/MWCNT-PhOMe and PLA/MWCNT-PhCOMe fibers, non-coated with the adhesion protein FN and let grow for 3 days. Cells' nuclei are stained with DAPI (blue channel), while the cytoskeletal actin fibers are stained red (Alexa Fluor 546 Phalloidin). In the green channel, vinculin is visible. White arrows in the merge images highlight the focal adhesion complex, while the yellow arrows point out the CNTs encapsulated into the polymeric fibers. Scale bar 20 μm. To better observe the focal adhesion arrangements in the proximity of CNTs aggregates, zoomed images are reported.

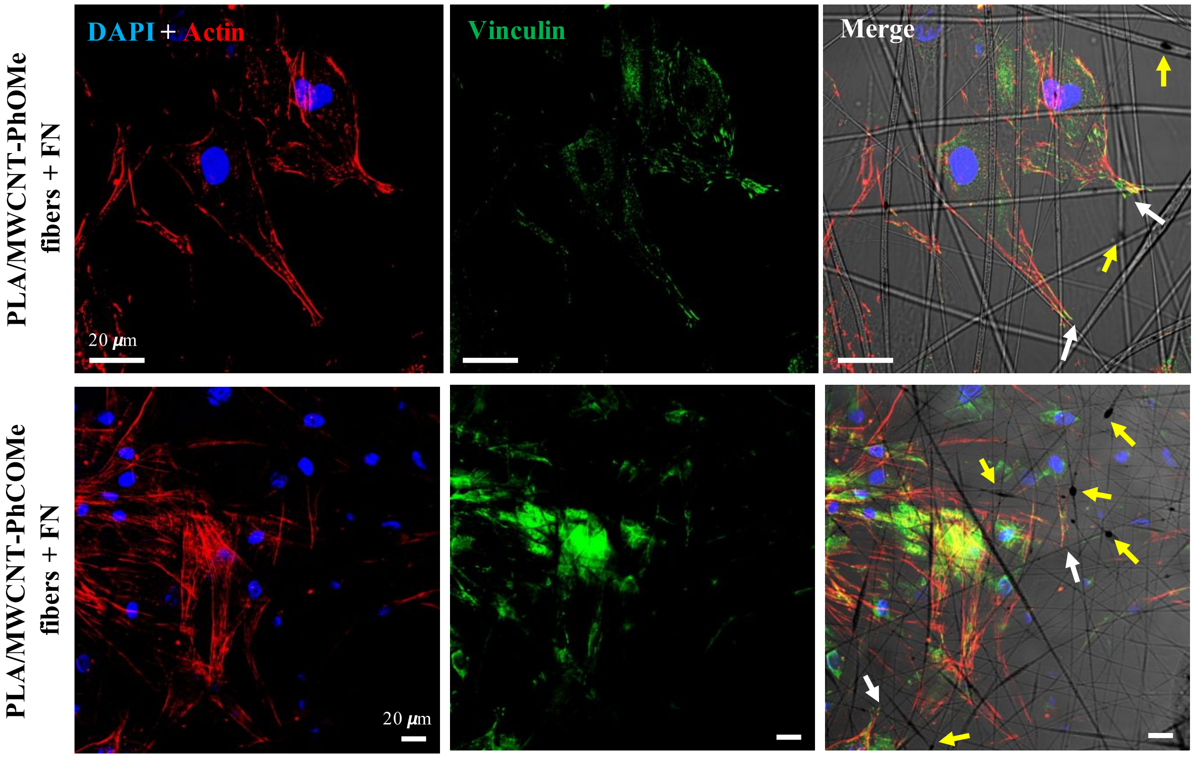

Figure S7: Effect of the fibrous matrices onto the fibroblasts focal adhesion complexes arrangements: confocal images of HDFa cells plated onto PLA/MWCNT-PhOMe and PLA/MWCNT-PhCOMe fibers, coated with the adhesion protein FN and let grow for 3 days. Cells' nuclei are stained with DAPI (blue channel), while the cytoskeletal actin fibers are stained red (Alexa Fluor 546 Phalloidin). In the green channel, vinculin is visible. White arrows in the merge images highlight the focal adhesion complex, while the yellow arrows point out the CNTs encapsulated into the polymeric fibers. Scale bar 20 μm.

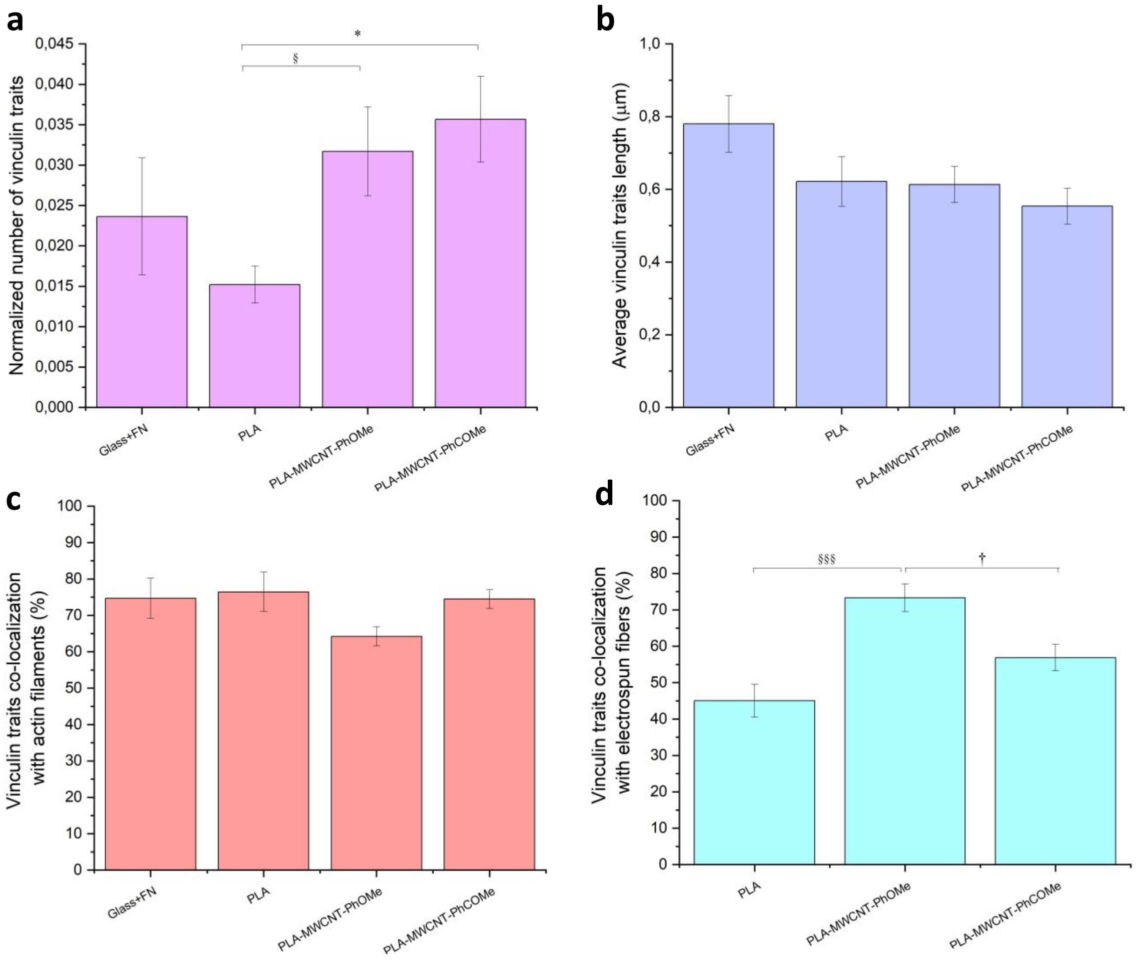

Sample	Average vinculin traits length (μm)	St. err.	Normalized vinculin traits number	St. err.
Glass + FN	0,780	0,078	0,024	0,007
PLA	0,622	0,068	0,015	0,002
PLA-MWCNT-PhOMe	0,613	0,049	0,032	0,006
PLA-MWCNT-PhCOMe	0,554	0,049	0,036	0,005

Sample	Co-localization with actin filaments (%)	St. err.	Co-localization with Eeectrospun fibers (%)	St. err.
Glass + FN	74,8	5,6	-	-
PLA	76,4	5,4	45,0	4,5
PLA-MWCNT-PhOMe	64,2	2,6	73,3	3,8
PLA-MWCNT-PhCOMe	74,5	2,6	56,9	3,6

Figure S8: Focal adhesion complex analysis: (a) Normalized number of vinculin traits (i.e. number of vinculin / cell area), (b) average length of vinculin traits (μm), and vinculin traits co-localization with either (c) the actin filaments or (d) the electrospun matrices (given as % values) of the cells plated onto the various substrates under study. Data are presented as mean value ± standard error. The significance was determined via ONE WAY ANOVA followed by Bonferroni's post-hoc test (parametric comparison) or a Kruskal-Wallis post-hoc test (non-parametric comparison) (*,†,§ for p value < 0,05; **,††,§§ for p value < 0,01; ***,†††,§§§ for p value < 0,001). More specifically, for the significance, (*) represents the comparison between the PLA sample and the PLA/MWCNT-PhCOMe substrate; (§) highlights the significance of the PLA-PhOMe substrate with the pristine PLA substrate; while (†) pinpoints that the statistical test was performed between the MWCNTs-containing samples.

The quantification of the normalized number of vinculin traits", i.e the number of vinculin traits divided by the cell area (graph a) indicates that PLA reduces the density of vinculin traits as compared to the control, whereas PLA/MWCNT-PhOMe and PLA/MWCNT-PhCOMe significantly promote the accumulation of fibroblasts vinculin traits, suggesting a more efficient ability to establish adhesion contacts. Of note, the average length of the vinculin traits (graph b) is comparable in all the samples, excluding the possibility of longer vinculin traits as an additional mechanism to promote cell adhesion. The comparable co-localization of vinculin with the actin filaments (graph c) in all samples, irrespective of the matrices composition, indicates that in all the conditions analyzed, the vinculin traits are functional, leading to the establishment of focal adhesion complexes. Moreover, the co-localization analysis of vinculin with the underneath substrates (graph d) to assess whether vinculin traits tend to aggregate in correspondence to the electrospun fibers, indicated higher values in the presence of PLA/MWCNT-PhOMe, further supporting the suitability of this functionalized scaffold for fibroblasts adhesion .

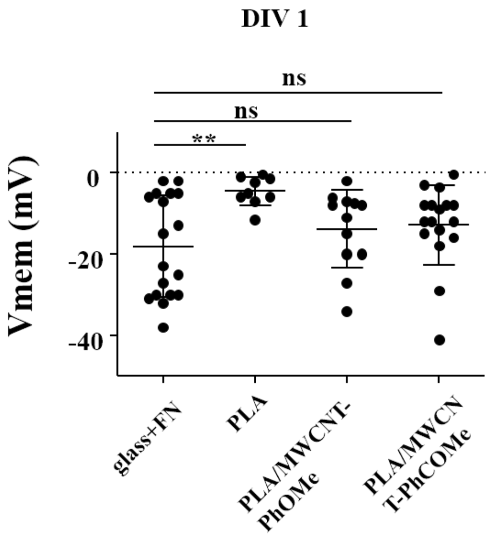

Figure S9: Characterization of the electrophysiological effects of the fibrous substrates onto primary fibroblasts: Membrane potential (mV) measurements recorded from HDFa cells at DIV1 on the various substrates (n=13-15 cells per condition, from 5 different preparations). V_{mem} values below 0 are considered hyperpolarized. ns, non-significant; ***, p<0.001; One-way ANOVA followed by Dunnett's post test.

Table S1: List of the primer acquired and relative gene sequence description.

Primer	Abbreviation	Amplicon Context Sequence	Amplicon Length (bp)
Collagen, type I, alpha 1	Col-α1	TCTTGGTCTCGTCACAGATCACGTCATCGCACAACACCTTGCCGTTGTCGCAGACGCAGATCCGGCAGGGCTCGGGTTTCCACACGTCTCGGTCATGGTACCTGAGGCCGTTCTGTACGCAGGTGATTGGTGGGATGTCTTCG	113
B-cell CLL/lymphoma 2	Bcl-2	TTGGACGAGGGGGTGTCTTCAATCACGCGGAACACTTGATTCTGGTGTTTCCCCCTTGGCATGAGATGCAGGAAATTTTTATTCCAATTCCTTTCGGATCTTTATTTCATGAGGCACGTTATTATTAGTAAGTATTGTTAATATCAGTCTACTTCCTCTGTGATGCTGAAAGGTT	145
BCL2-associated X protein	Bax	GCACCAAGGTGCCGGAACTGATCAGAACCATCATGGGCTGGACATTGGACTTCCTCCGGGAGCGGCTGTTGGGCTGGATCCAAGACCAGGGTGGTTGGGTG	71
Glyceraldehyde-3-phosphate dehydrogenase	GAPDH	GTATGACAACGAATTTGGCTACAGCAACAGGGTGGTGGACCTCATGGCCCACATGGCCTCCAAGGAGTAAGACCCCTGGACCACCAGCCCCAGCAAGAGCACAAGAGGAAGAGAGAGACCCTCACTGCTGGGGAGTCCCTGCCACAC	117
